# Phenotypic and microRNA transcriptomic profiling of the MDA-MB-231 spheroid-enriched CSCs with comparison of MCF-7 microRNA profiling dataset

**DOI:** 10.7717/peerj.3551

**Published:** 2017-07-13

**Authors:** Lily Boo, Wan Yong Ho, Norlaily Mohd Ali, Swee Keong Yeap, Huynh Ky, Kok Gan Chan, Wai Fong Yin, Dilan Amila Satharasinghe, Woan Charn Liew, Sheau Wei Tan, Soon Keng Cheong, Han Kiat Ong

**Affiliations:** 1 Faculty of Medicine and Health Sciences, Universiti Tunku Abdul Rahman, Cheras, Selangor, Malaysia; 2 Department of Biomedical Sciences, University of Nottingham, Semenyih, Selangor, Malaysia; 3 Xiamen University Malaysia, Sepang, Selangor, Malaysia; 4 Department of Genetics and Plant Breeding, Cantho University, Cantho, Vietnam; 5 Division of Genetics and Molecular Biology, University of Malaya, Kuala Lumpur, Malaysia; 6 Faculty of Veterinary Medicine and Animal Science, University of Peradeniya, Peradeniya, Central, Sri Lanka; 7 Institute of Bioscience, Universiti Putra Malaysia, UPM Serdang, Selangor, Malaysia; 8 Cryocord Sdn Bhd, Cyberjaya, Selangor, Malaysia

**Keywords:** Breast cancer, Cancer stem cells, MDA-MB-231, Next generation sequencing, Triple-negative breast cancer cells, MicroRNA, Spheroid culture

## Abstract

Breast cancer spheroids have been widely used as *in vitro* models of cancer stem cells (CSCs), yet little is known about their phenotypic characteristics and microRNAs (miRNAs) expression profiles. The objectives of this research were to evaluate the phenotypic characteristics of MDA-MB-231 spheroid-enriched cells for their CSCs properties and also to determine their miRNAs expression profile. Similar to our previously published MCF-7 spheroid, MDA-MB-231 spheroid also showed typical CSCs characteristics namely self-renewability, expression of putative CSCs-related surface markers and enhancement of drug resistance. From the miRNA profile, miR-15b, miR-34a, miR-148a, miR-628 and miR-196b were shown to be involved in CSCs-associated signalling pathways in both models of spheroids, which highlights the involvement of these miRNAs in maintaining the CSCs features. In addition, unique clusters of miRNAs namely miR-205, miR-181a and miR-204 were found in basal-like spheroid whereas miR-125, miR-760, miR-30c and miR-136 were identified in luminal-like spheroid. Our results highlight the roles of miRNAs as well as novel perspectives of the relevant pathways underlying spheroid-enriched CSCs in breast cancer.

## Introduction

There has been a substantial amount of evidence indicating that tumours contain a group of self-renewing cancer cells known as cancer stem cells (CSCs) which are responsible for tumour initiation, progression, and metastasis (Chen, Huang & Chen, 2013). Cancer therapies are clinically focused on eradicating the overall cancer cell population, rather than the CSCs population, which often resulted in high recurrence as CSCs have shown to be highly resistant to chemotherapy. Therefore, understanding the underlying biology and molecular mechanisms of CSCs would be of paramount importance in revolutionizing cancer therapies.

Over the past years, the challenge in CSCs research is isolating a pure population of CSCs as compelling evidences have shown that CSCs were estimated to be only 0.001–0.1% (Visvader & Lindeman, 2008) of isolated tissues, suggesting the heterogeneous complexity of the tumours. Although numerous methods have been proposed in order to isolate and expand the cells to the sufficient amount feasible for research use, these methods comes with their own challenges. The most common approach would be cell sorting using flow cytometry technology based on cell surface markers specifically targeting the CSCs populations from the rest of the non-CSCs populations (Duan et al., 2013). Nevertheless, identification of CSCs based on surface markers is difficult as there is not a single marker which would be sensitive and specific enough to define the actual CSCs populations. The use of ALDEFLOUR assay, a stem cell marker based on enzymatic activity of ALDH1 was proposed to be the next reliable tool to isolate CSCs from tumour(Croker et al., 2009). Nonetheless, this approach is not recommended as a standalone assay as it requires other marker combinations to further separate the CSCs populations from the tumour. Taken together, more useful and effective assays are needed to culture CSCs population subtypes.

Previous studies have demonstrated the successful application of spheroid formation technique in isolating, enriching, and expanding putative CSCs subset from a range of cancer cell lines and cancer tissues which provided a potential model towards the development of targeted therapies to study CSCs *in vitro* (Cao et al., 2011; Fang et al., 2005). The CSCs population enriched in serum-free culture condition favoured their expansion while the rest of non-CSCs population undergo anoikis. The significance of enriching CSCs in multicellular spheroids has been supported by a pre-clinical study that indicated such spheroid-enriched cells as a feasible CSCs model to elucidate the chemoprevention properties of sulforaphane in breast cancer treatment (Li et al., 2010). In breast cancer research, this technique appeared to be more reliable and useful tool to select and expand CSCs populations in manner sufficient for its use in functional studies. Additionally, the conventional two-dimensional (2D) monolayer cultures commonly used to maintain and expand cancer cells have often showed loss of tumour function (Kim, Stein & OH, 2004) whereas three-dimensional (3D) culture that recapitulates the *in vivo* solid tumour biology has been the more favourable culture choice of demonstrating the overall features of the cancer cells (Ho et al., 2012; Pickl & Ries, 2008). However, despite the numerous functional studies on the response of these spheroid models in drug resistance therapies, little is known about the underlying mechanisms of the breast cancer spheroid CSCs.

Breast cancer can be divided into a few subtypes with regards to their molecular characteristics in which luminal and basal type being the two most commonly studied. MCF-7 cells, a luminal type is non-metastatic whereas MDA-MB-231 cells, which lacks of the three breast receptors (ER, PR and HER2), are regarded as highly aggressive (Kao et al., 2009). Cell lines are commonly used to model breast cancer *in vivo* as they are easily accessible, reliable, and less problematic compared to the primary culture of tumours. Moreover, transcriptomic features of breast cancer cell lines were found to be similar to their respective tumours, suggesting the clinical usage of these cell lines in breast cancer research (Vincent, Findlay & Postovit, 2015).

MicroRNAs (miRNAs), a class of short noncoding RNAs that has been known as an important class of molecules regulating gene expressions (Stefanie, Eric & Caldas, 2008). The gene regulatory molecules are responsible for a wide range of diseases including oncogenesis and are therefore proposed to be promising biomarkers or act as therapeutic targets (Mishra, 2014). Consequently, miRNAs profiling has been carried out extensively to identify cancer-specific miRNAs signatures in various cancers (Murakami et al., 2014; Nygaard et al., 2009; Wyman et al., 2009). In our recent published work, we have identified the miRNAs of luminal MCF-7 spheroid-enriched CSCs, with some miRNAs which have not been previously associated with breast cancer (Boo et al., 2016). In this work, we first showed that the basal cell line, MDA-MB-231 formed spheroids and demonstrated different CSCs features compared to MCF-7 spheroids. MiRNA-NGS analysis on the MDA-MB-231 spheroids were also conducted and compared with the miRNA profiling against MCF-7 spheroids to investigate the roles of miRNAs in the spheroid-enriched CSCs models derived from these two breast cancer subtypes. Though MCF-7 and MDA-MB-231 belong to two distinct subtypes, they could be sharing some similar miRNAs cluster possibly linking to the molecular characteristics of spheroid CSCs. We then focused our attention on their gene annotations of the differentially expressed miRNAs-targets and pathways inferred using bioinformatics tools. Collectively, this study provides potentially new miRNAs markers to target breast CSCs via comparison of the miRNAs profiling of the two breast spheroid-enriched subtypes.

## Materials and Methods

### Cell culture, spheroid formation assay and SEM analysis of MDA-MB-231

The estrogen-independent human breast adenocarcinoma MDA-MB-231 was purchased from American Type Culture Collection (ATCC) (Catalog no. HTB-26). The cell line was maintained as monolayer at 37 °C under a 5% humidified CO_2_ atmosphere in Roswell Park Memorial Institute (RPMI)-1640 medium supplemented with 10% heat-inactivated fetal bovine serum. Spheroid cells were generated in a serum-free environment according to our published method (Boo et al., 2016). To analyse the surface ultrastructures of the generated spheroids, SEM analysis was performed following the standard sample preparation protocol but with additional centrifugation at each step to minimise sample loss. The specimens were then mounted, critically point dried and sputtered gold coated before viewed using a SEM (JSM-6400; JEOL, Tokyo, Japan).

### Secondary spheroids and single cell formation assay

The formed spheroids were then collected, pooled by centrifugation, and then enzymatically dissociated into single cells with Accutase (GIBCO, Gaithersburg, MD, USA). The resulted single cells were then sieved through a 40 µm strainer before seeded at 200 cells per well in96-well agar-coated dishes in 100 µL of serum-free growth medium. The sphere forming efficiency (SFE) was then evaluated based on the sphere formation rate over a course of 14 days. Self-renewal ability of the spheroids was also further tested by limiting dilution assay whereby single cells were plated into an ultra-low attachment 96-well plate (Corning, NY, USA). The process of the secondary spheroids formed from the single cell was recorded using a phase-contrast microscope.

### Expression of CSCs surface markers and ALDH activity

The expression of the surface markers CD44+/CD24− was measured using anti-human CD44 conjugated with FITC and anti-human CD24 conjugated with PE (Mitenyl Biotech, Bisley, Surrey, UK). A total of 1 × 10^6^ singlecells was collected by trypsinisation and filtered using a 70 µm membrane filter prior to specific antibodies staining in accordance to the standard protocol. To measure ALDH activity (Stem Cell Technologies, Vancouver, Canada), single cells were incubated with the activated Aldeflour reagent for 45 min at 37 °C. The expression of the CSCs surface markers and ALDH activity were acquired using a FACS Calibur (BD Biosciences, San Jose, CA, USA).

### Immunofluorescence characterization

To stain the monolayer, parental cells were trypsinised and grew as monolayer on a chamber slide. Spheroids, on the other hand, were washed and harvested by centrifugation. Both monolayer and spheroid cells were then fixed in cold paraformaldehyde for 5 min before incubated with monoclonal antibodies anti-human conjugated with fluorescent dyes. The antibodies used were CD24-PE, CD44-FITC, CD49f-FITC, Sox2-PE, Nanog-AF, and ALDH-FITC. Staining was performed by incubating the antibodies overnight at 4 °C. Before viewing and capturing the images, the samples were stained with a nuclear stain DAPI (GIBCO, Gaithersburg, MD, USA).

### MTT (3-[4, 5-dimethylthiazol-2-yl]-2, 5-diphenyltetrazolium) assay

To determine the drug resistancy of spheroid cells, cytotoxicity assay was in accordance to the previously published method on intact spheroids (3D condition) (Ho et al., 2012). Conversely, the MTT assay for monolayer cells and dissociated spheroids (2D condition) followed the standard cytotoxicity protocol (Mosmann, 1983). Three chemotherapeutic drugs (tamoxifen, cisplatin and doxorubicin) were serially diluted into different concentrations and the cytotoxicity was determined by the resulted absorbance values taken at 570 nm. To calculate the percentage of cell viability, the mean absorbance value of the treated cells was measured and compared with the control wells. The IC_50_ values for the spheroids and parental cells subjected to different drugs were then obtained from the dose–response graphs.

### Cell proliferation assay

Cell proliferation assay of the spheroid and parental cells was performed using alamar Blue Cell Viability Assay Reagent (Thermo Scientific, Waltham, MA, USA). Briefly, the cultures were incubated with 10% of alamar Blue reagent of the total volume of the medium in the well for 4 h. The assay was carried at specific time intervals for three weeks. The cell proliferation based on the value of absorbance at 570 nm and 600 nm was measured using a microplate reader. To calculate the rate of cell proliferation, the percentage difference in the reduction of alamar Blue reagent between the treated and control samples were determined (Rampersad, 2012).

### *In vitro* scratch assay

The wounds were initiated using a pipette tip scratched across the centre in a perpendicular manner of the well. The cells were allowed to grow for 24 h. The gap distance of the wound was then qualitatively captured using an inverted microscope (Nikon, Tokyo, Japan) at 6, 12 and 24 h post-wound. The mean percentage of cell migration rate to close up the wounds at different time points were then quantified using Image J software (Schneider et al., 2012).

### Invasion and migration assay

The invasion assay was carried out by starving the cells in serum-depleted medium a day before the assay. The next day, the cells were harvested, suspended into single-cell and placed onto the top chambers of transwell inserts (BD Biosciences, San Jose, CA, USA) coated with Matrigel. The inserts placed at the top of a 24-well were incubated in a serum-depleted medium whereas medium containing serum was placed at the bottom of the well. Detection of cell invasion was determined after 72 h by fixing and staining the inserts with 0.5% crystal violet solution. The dye was then later extracted using 30% acetic acid before the absorbance values at 590 nm was measured. To calculate the percentage of the invaded cells, the absorbance values of the samples divided by the absorbance of the control were determined. For migration assay, similar steps were performed but with the absence of Matrigel coating.

### MiRNAs extraction and quality check

Total RNAs including microRNAs were isolated from the three parental and three spheroids samples using a miRNeasy kit (Exiqon, Vedbæk, Denmark) following the manufacturer’s protocol. To ensure only good quality of RNAs were used, RNA concentration was measured using Qubit 2.0 Fluorometer (Invitrogen, Carlsbad, CA, USA) and RNA integrity was determined with Agilent 2100 Bioanalyzer (Applied Biosystems, Foster City, CA, USA). Only samples with concentration >600 ng/µL and intact RNA (RIN>8) were used to prepare the miRNA libraries.

### Next generation sequencing (NGS) of miRNAs

Approximately 3 µg of total RNA from the samples was taken into small RNA library preparation protocol using TruSeq Small RNA Sample Prep Kit (Illumina, San Diego, CA, USA) in accordance to the manufacturer’s protocol. Briefly, the miRNA molecules from different samples were ligated to 5′  and 3′  adaptors sequentially prior to be reverse-transcribed to cDNA followed by PCR amplification. Index sequences made of six-base combinations were then incorporated into the PCR products before they were pooled and run on a 6% PAGE gel. The final libraries resulted from the gel purification were then validated using Bioanalyzer HS-DNA chips before they were normalised to a final concentration of 2 nM and sequenced in the Illumina’s HiSeq Run.

### Small RNA bioinformatics analysis

Analysis of the raw NGS data included the trimming, filtering, and cleaning up the contaminated reads was performed using CLC Genomics Workbench 7.0. The sequences with shorter read length of 17 and more than 27 were removed. The normalised trimmed read lengths were aligned to the Ensembl human database (Homo sapiens GRCh 37.57) and known miRNAs database (miRBase-19) using the Illumina 1.8 pipeline. The processed miRNAs data was then visually assessed using quality control plots. The differential profiling of genome-wide miRNAs between the spheroid and parental was then compared using Kal’s *Z*-test and the resulting *p*-values were background corrected using Benjamini–Hochberg method. The statistically significant differentially expressed miRNAs (FC >  2, *P* < 0.05) of the spheroid cells in relative to parental cells were then generated.

### Comparison of miRNAs profiling between MDA-MB-231 and MCF-7 spheroid-enriched CSCs

Next, the miRNAs transcriptome profiles obtained in this study was used to compare to the previously published work on miRNAs transcriptome profiling on MCF-7 spheroid enriched CSCs (GSE68246). The purpose of the comparison is to identify the potential miRNAs that could be found in spheroid-enriched CSCs cells of different breast cancer cell subtypes and to possibly elucidate the mechanisms attributed to the two spheroid models. A Venn diagram was constructed to analyse the differentially or the commonly expressed miRNAs between the two cell types. In order to elucidate the functional roles of the miRNAs, the online Database for Annotation, Visualization, and Integrated Discovery (DAVID) program (http://david.abcc.ncifcrf.gov/tools.jsp) (Huang, Sherman & Lempicki, 2008; Huang, Sherman & Lempicki, 2009) was used for functional annotations and enriched pathways using the KEGG. For each GO annotation, a *p*-value is calculated and sorted based on their *p*-values. To identify the most specific GO annotation for the groups of analysed genes, the *p*-values were ranked and the lowest *p*-values indicate the most significant term. Identification of the GO of the commonly deregulated miRNAs and those uniquely expressed miRNAs between the two spheroid cells was performed.

### Interaction networks between miRNAs-target genes and the functional analysis of target genes

MicroRNA is known to targets more than one gene, and one gene is known to be targeted by more than one miRNA. To visualise the complex underlying networks between the miRNA and their target genes, three prediction programs, namely, miRTarBase_hsa_r4.4 (http://mirtarbase.mbc.nctu.edu.tw/), TargetScan Homo sapiens version 6.2 (http://www.targetscan.org/), and MicroCosm v5 Homo sapiens (http://www.ebi.ac.uk/enright-srv/microcosm/htdocs/targets/v5/) available in Cytoscape V3.3, (http://www.cytoscape.org/) were used. Enrichment analysis of the predicted miRNAs targets were then performed using web-based tool DAVID as mentioned in the previous section. *P*-values <0.05 were considered to be statistically significant for the functional analysis.

### qRT-PCR

To validate the NGS data, SYBR green qRT-PCR was performed using Exiqon SYBR green master mix. In brief, 100 ng of total RNA with retention of miRNAs was polyadenylated at their 3′  ends and reverse-transcribed into cDNA. The PCR amplification was then carried out following standard protocol. Primer sequences for qRT-PCR are listed in Data S6. Quantitative PCR was carried out using CFX96 Real-time PCR Detection System (Bio-Rad Laboratories, Hercules, CA, USA). Normalization was done using the average values of miR-200a and miR-19 as the endogenous controls evaluated using geNORM algorithms.

### Reversal of spheroid culture

The generated spheroids were subjected to monolayer culture to induce the growth of reversal spheroid cells. Spheroids cells were transferred into a 6-well culture dish maintained in serum-supplemented growth medium, and were allowed to migrate out as monolayer cells. The characteristics of the reversal spheroid cells were determined using a phase-contrast microscope and the miRNAs levels were further validated using SYBR green qRT-PCR.

### Statistical analysis

Experiments were performed in three replicates unless otherwise stated. All statistical analyses were performed using independent sample one-way analysis of variance (ANOVA) and student independent *t*-test via SPSS V17 software. Data were considered statistically significant at a probability level of *P*-value <0.05.

## Results

### Generation of spheroid cells from MDA-MB-231 breast cancer cell line

**Figure 1:**
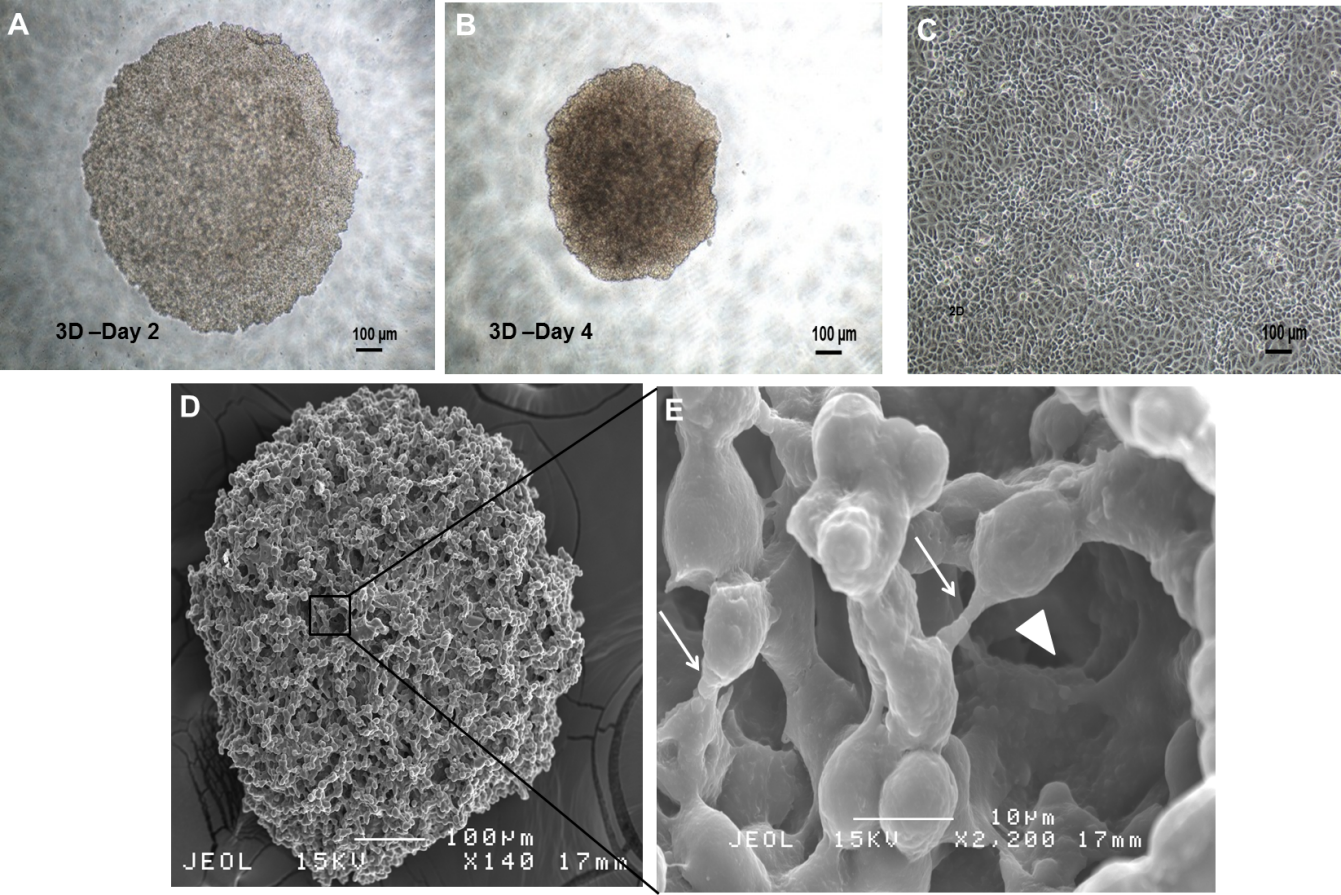
Spheroid and monolayer cells from estrogen-independent human breast adenocarcinoma cell line MDA-MB-231. (A, B) Spheroid cells organised themselves into a compact and rigid three-dimensional structure 96 h post-culture in serum-free environment (magnification: 4×, scale bar; 100 µm). (C) Parental cells cultured in two-dimensional monolayer condition displayed as spindle shaped cells and adherent in nature (magnification: 4×, scale bar; 100 µm). (D) Ultrastructure analysis of spheroid cell coincided with clumping of cells to one another, with preservation of the overall structure of the spheroidal architecture (magnification: 140×, scale bar; 100 µm). (E) Higher SEM magnification showed the presence of cell–cell junctions (arrows) within the spheroid that are responsible for maintaining strong cell–cell contact (magnification: 2,200×, scale bar; 10 µm). Micropores indicated by ‘arrowheads’ allowed the exchange of nutrients across the inner layer of the spheroid with their surroundings.

**Figure 2:**
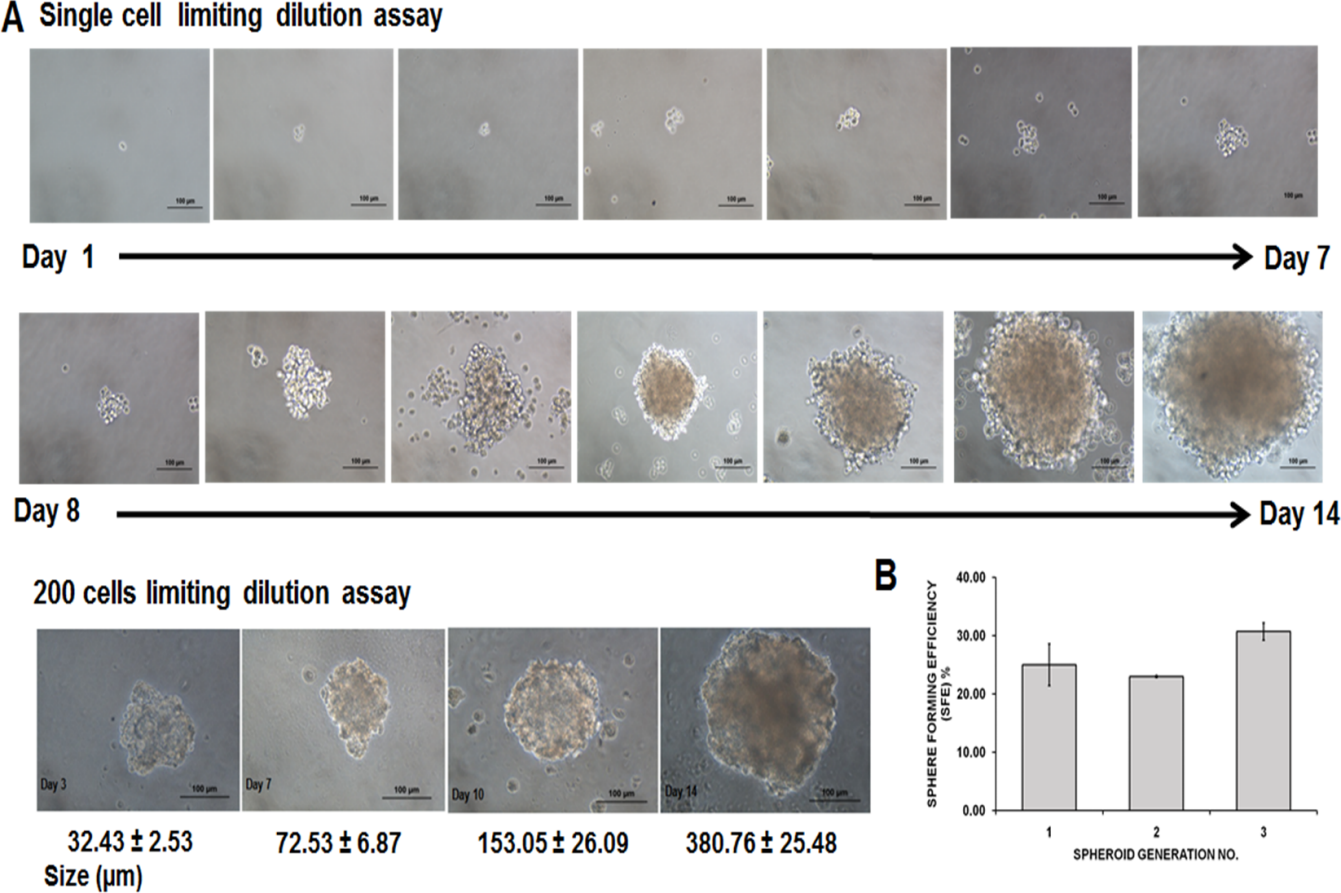
Secondary MDA-MB-231 spheroids formed using limited dilution techniques at single cell seeding and 200 cells/well. (A) Generation of secondary spheroids from a single cell acquired microscopically for 14 days (magnification: 20×, scale bar; 100 µm). Secondary spheroids showed increased size spheroids from day 3 to day 14 (magnification: 20×, scale bar; 100 µm). Size of the spheroids and images are representative of three biological replicates. (B) Sphere-forming assay of secondary spheroids of MDA-MB-231 cell lines. SFE was counted from first to third generation. Data are based on the mean percentages of the number of spheres formed within a culture relative to the initial cell seeding number (means ± SD, *n* = 3)

MDA-MB-231 cells formed three-dimensional spheroids within 24–48 h in the serum-free and anchorage-independent culture system. In contrary, the parental remained as monolayer cells (Figs. 1A–1C). The spheroids were mostly homogenous in size after being cultured for 96 h. Cell count revealed a reduction of cell number on day 4 (initial number of cells: 5.00 × 10^4^ cells; spheroid day 4: 4.76 ± 0.08 × 10^4^). Ultrastructure images captured using Scanning Electron Microscope (SEM) at 140X magnification demonstrated that the spheroids were made up of cell aggregation (Fig. 1D). Higher magnification at 2,200× showed that the cells within the intact spheroid were held closely forming a spherical morphology and cell to cell junctions were visible surrounding the cells resulted in a relatively compact structure (Fig. 1E). Their self-renewability of the dissociated cells was measured by their spheroid ability to increase in size over a course of 14 days and the ability of the single cell to be clonally expanded (Fig. 2A). The resulted spheres were then harvested, dissociated into single cells, replated on the ultra-low plates and this process was repeated twice to assess the sphere-forming efficiency (SFE) of the spheroid cells (Fig. 2B). The capacity of the MDA-MB-231 spheroid cells to be serially passaged demonstrated the self-renewing ability of the cells which is a common property of cells enriched with CSCs properties.

### Presence of subpopulations of cells CD44+/CD24−/low and ALDH+ in spheroid MDA-MB-231

**Figure 3:**
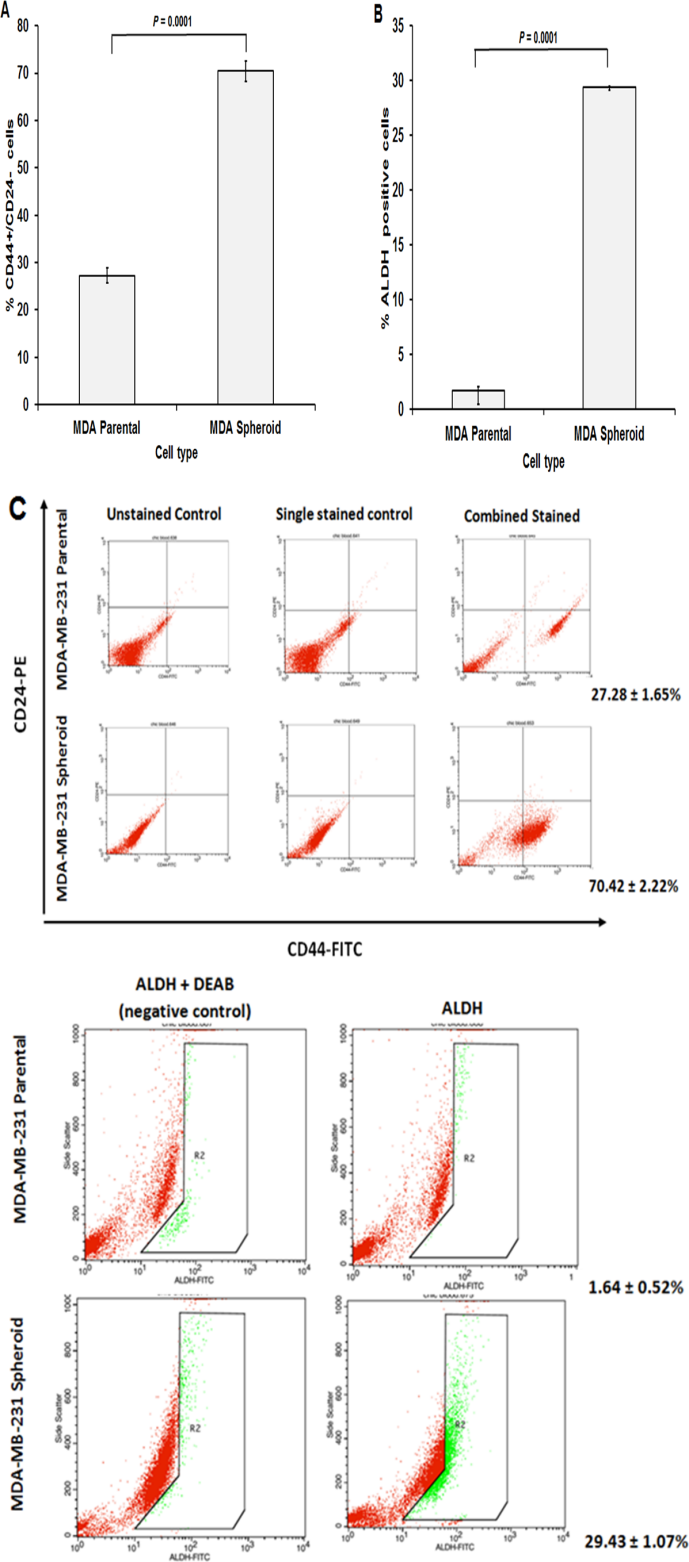
Cancer stem cells surface markers and ALDH activity of MDA-MB-231 spheroids assessed by flow cytometry analysis. (A) Spheroid cells exhibited a significantly higher percentage of CD44+/CD24− cells populations (70.42 ± 2.22%) compared to that of the parental cells (27.28 ± 1.65%). (B) ALDH activity also exhibited an increased in spheroid cells with 29.43 ± 1.07% compared to parental cells 1.64 ± 0.52%. (C) Representative flow cytometric dot plots were shown whereby R2 is the region of ALDH-positive cells.

Breast CSCs have been shown to express CD44+/CD24−/low phenotype (Ricardo et al., 2011). Increased expression of ALDH activity has also been linked with cells with CSCs-like properties (Ginestier et al., 2007). We found that the proportion of CD44+/CD24−/low cells were 2.5-fold significantly higher in the spheroid population (70.42 ± 2.22%) than its parental cells counterparts (27.28 ± 1.65%) (mean ± SD; *n* = 3) (Fig. 3A). Meanwhile, spheroid cells showed a higher ALDH activity (29.43 ± 1.07%) compared to the ALDH activity of the parental cells (1.64 ± 0.52%) (mean ± SD; *n* = 3) (Fig. 3B). The dot plots in Fig. 3C show the distribution of the cells expressing CD44+/CD24−/low and ALDH +ve. These results suggest that spheroid cells in serum-free condition were enriched with CD44+/CD24−/low subpopulation and ALDH +ve, showing another characteristic of CSCs.

### Spheroid MDA-MB-231 cells exhibit stem cell-like markers and ALDH activity

To investigate the stemness characteristics, the immunofluorescence staining were tested on the spheroids and their parental cells Data S1. It was found that the spheroid cells were positively stained with surface markers CD44 and CD49f and intracellular markers Sox 2 and Nanog with decreased or non-detectable expression in control cells. Additionally, a significantly higher ALDH activity, another marker for stem/progenitor cell was found in the spheroid cells compared to control. Besides, the expression of the stem cell-like markers was also observed in secondary spheroids, indicating the preservation of the stem cell features of the spheroid cells. Taken together, these data indicated that the generated spheroids possessed CSCs characteristics of which were not observed on the parental cells.

### Spheroid MDA-MB-231 cells are resistant to chemotherapeutics drugs

The CSCs characteristic of the MDA-MB-231 spheroids was further confirmed for the increased resistance to chemotherapeutics drugs. We compared the drug resistance between the spheroid cells in 3D and 2D culture conditions to the parental cells using MTT assay.

Overall, the MDA-MB-231 spheroid cultured in 3D or 2D conditions demonstrated higher resistance to the three tested drugs compared to the parental cells (Table 1). The drug inhibitory concentration (IC_50_) under 100 µg serially diluted drugs for 3D spheroids relative to parental cells were 3.67-fold, 11.24-fold, and 9.00-fold higher for tamoxifen, doxorubicin and cisplatin, respectively. Similarly, the spheroids dissociated cells (2D condition) were 3.63-fold, 16.87-fold, and 2.30-fold higher resistance to the same chemotherapeutics drugs in comparison to its parental counterpart. Increased drug resistance in spheroid cells could be attributed by the higher proportions of CSCs in the enriched cells compared to monolayer cells.

**Table 1:** Determination of the IC_50_values of different chemotherapeutic drugs (tamoxifen, doxorubicin, and cisplatin) on spheroid (3D and 2D conditions) and parental cells of MDA-MB-231. Serially diluted drugs were added to the cells and MTT assays were performed at 96 h. The 50% inhibition rates of the drugs against the cells were determined from the dose–response curves. Data are expressed as the means value of three experiments replicated with standard deviations (SD). Asterix indicates statistically significant values at *p* < 0.05. IC_50_ values of previously published study were also incorporated in the table.

		IC_50_(µM)		
		Tamoxifen	Doxorubicin	Cisplatin
Current study (basal-like derived cells)	MDA-MB-231 Parental	10.28 ± 0.27	0.719 ± 0.13	1.74 ± 0.38
	MDA-MB-231 Spheroid (3D)	37.72 ± 5.31^*^	8.083 ± 2.44^*^	15.67 ± 1.71^*^
	MDA-MB-231 Spheroid (2D)	37.34 ± 3.82^*^	12.13 ± 3.61^*^	4.00 ± 0.96^*^
Published study^a^ (luminal-like derived cells)	MCF-7 Parental	20.57 ± 2.25	1.00 ± 0.37	2.54 ± 0.44
	MCF-7 Spheroid (3D)	60.73 ± 7.39^*^	18.82 ± 3.05^*^	13.61 ± 3.54^*^
	MCF-7 Spheroid (2D)	47.62 ± 6.83^*^	13.25 ± 3.69^*^	13.68 ± 3.92^*^

**Notes.**

a Adapted from our related published work.

### Different cell behaviour in spheroid MDA-MB-231 cells

**Figure 4:**
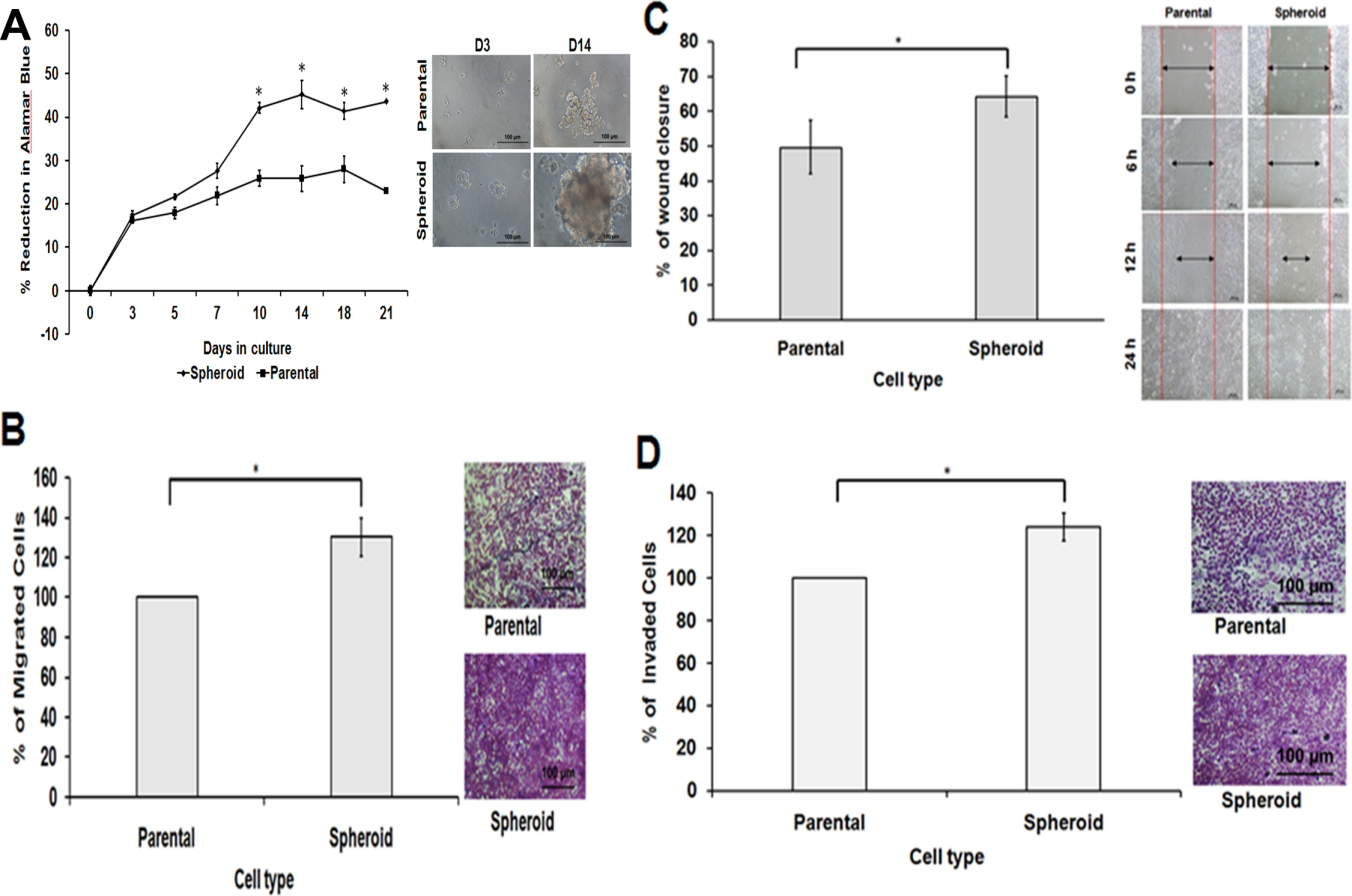
Spheroid cells demonstrated increased cell proliferation, migration, wound healing ability and invasion capacity relative to parental cells. (A) Comparison of cell proliferation ability of the spheroid cells monitored using Alamar Blue assay. Determined by the reduction of Alamar Blue, spheroid cells showed significantly higher cell proliferation than dissociated parental cells when cultured in the same seeding number and condition. (B, C and D) Bar charts show higher cell migration, wound healing and invasion abilities in spheroid cells compared to their parental controls. Cell migration and invasion assays were measured by extracting the resulted stained cells and the absorbance of the dye colours were quantified. On the other hand, in wound healing assay, a gap was created and the cells’ proliferative ability to close the gaps were measured and recorded. The wound healing process of the spheroid and parental cells were taken at 0, 6, 12 and 24 h post-wound initiation.

In cell proliferation assay, disaggregated spheroid cells showed a higher proliferation rate compared to the parental cells (Fig. 4A). This was further supported by the migration and wound healing assays where spheroid cells moved faster from the edge of the scratch to close up the wound (Fig. 4C). On the other hand, spheroid cells also demonstrated increase in cell invasion ability, as there were a higher cell number passing through the membrane filter (Figs. 4B and 4D) compared to the control. Spheroids are known to be enriched with CSCs subpopulations; therefore, different cell behaviour in terms of their cell proliferation, migration and invasion were noted in spheroid cells.

### MiRNAs sequencing analysis of MDA-MB-231 spheroids compared with parental cells

The miRNAs profiling were found to be significantly different in spheroid relative to parental cells. On average, a total of 22,096,678 and 26,517,408 reads were obtained for parental and spheroids, respectively with a quality score (Q30) of 93.3%. After trimming the 5′  and 3′  adaptor sequences, the remaining effective reads were mapped to the sequence data in the miRBase19 microRNA Sequence Database of which a total of 8,435,715 and 10,979,357 reads were obtained from parental and spheroids, respectively. The average length of the detected sequences was 22 nucleotides (Data S2) for both parental and spheroids. Among these reads, 54,042 corresponding to 6,656,191 reads and 29,260 corresponding to 1,764,277 reads were matched to known miRNAs sequences in parental and spheroids, respectively. The trimmed reads revealed that there were an average of 39.6% and 40.5% of spheroids miRNAs that corresponded to the miRBase-19 (Homo sapiens) database. The remaining sequences were found to be matched to the non-coding database (Homo Sapiens GRCh 37.57) with 60.4% in parental and 59.5% in spheroid cells. The high throughput miRNA sequencing data can be accessed at the National Center for Biotechnology Information (NCBI) with the reference number GSE75396. The summary statistics of the miRNA libraries of spheroid and parental cells can be found in Data S3. As shown in Data S4, the two groups were successfully segregated using the hierarchical cluster analysis and samples with similar patterns of expression of miRNAs studies were clustered together as indicated by the heatmap. Visualisation of the miRNAs expression dataset by principal component analysis (PCA) revealed a low level of inter-samples variations of the biological replicates of spheroids and parental. Volcano plot filtering was also performed to identify the significant levels of differentially expressed miRNAs between the two groups. A total of 69 significantly differentially expressed miRNAs with fold change >2 were found in the spheroid cells relative to the parental cells. Out of these 69 miRNAs, 33 were found to be up-regulated in spheroid cells whereas 36 were down-regulated (Data S5), suggesting there were distinct miRNAs profiling in spheroid relative to parental cells.

### Comparison of miRNAs profiling between spheroid-enriched CSCs of two breast cancer subtypes

**Figure 5:**
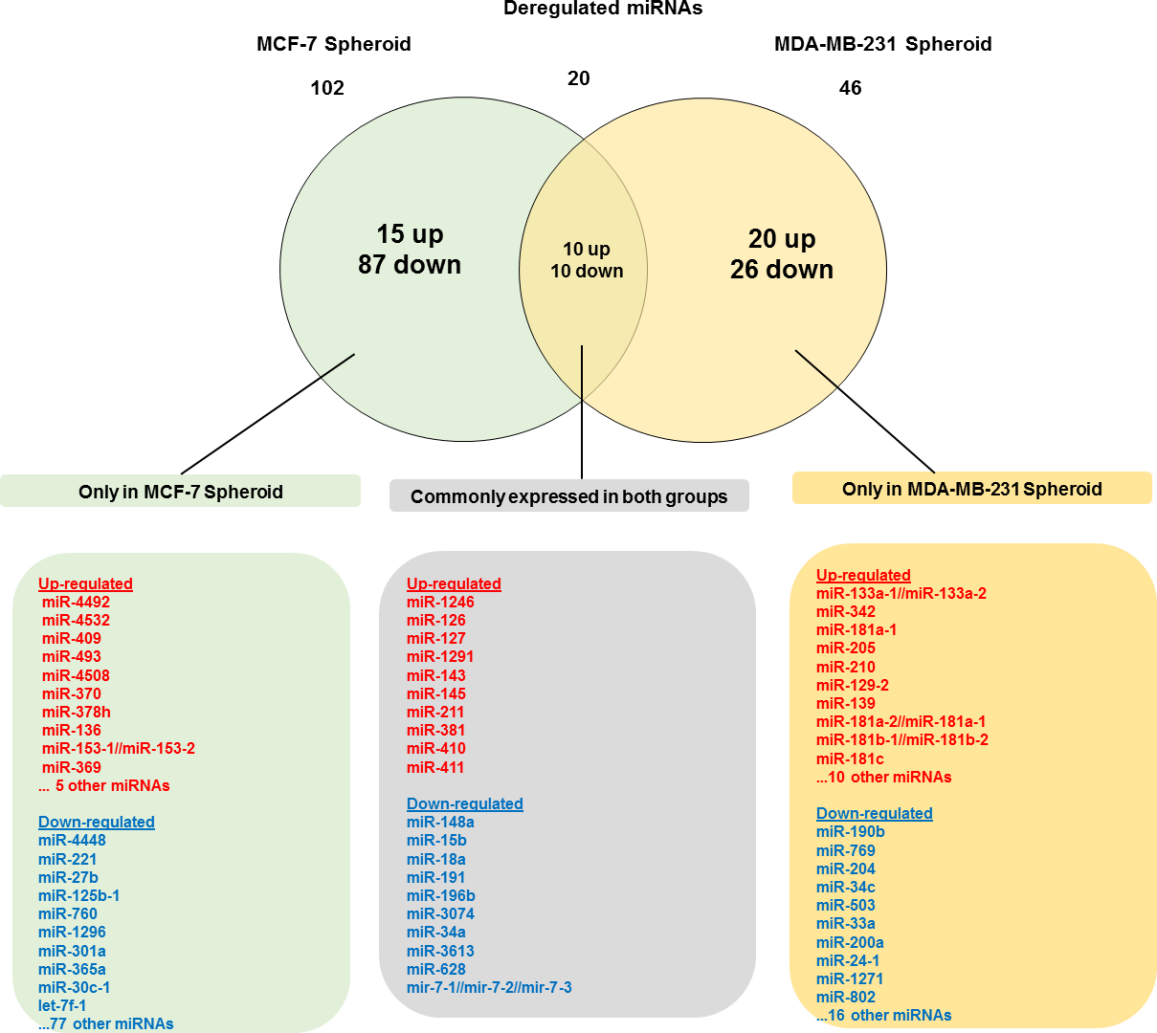
Venn diagram analysis showing the number of differentially expressed miRNAs that are unique and commonly expressed in MCF-7 and MDA-MB-231 spheroids. A total of 20 commonly deregulated miRNAs were found in both spheroids.

**Figure 6:**
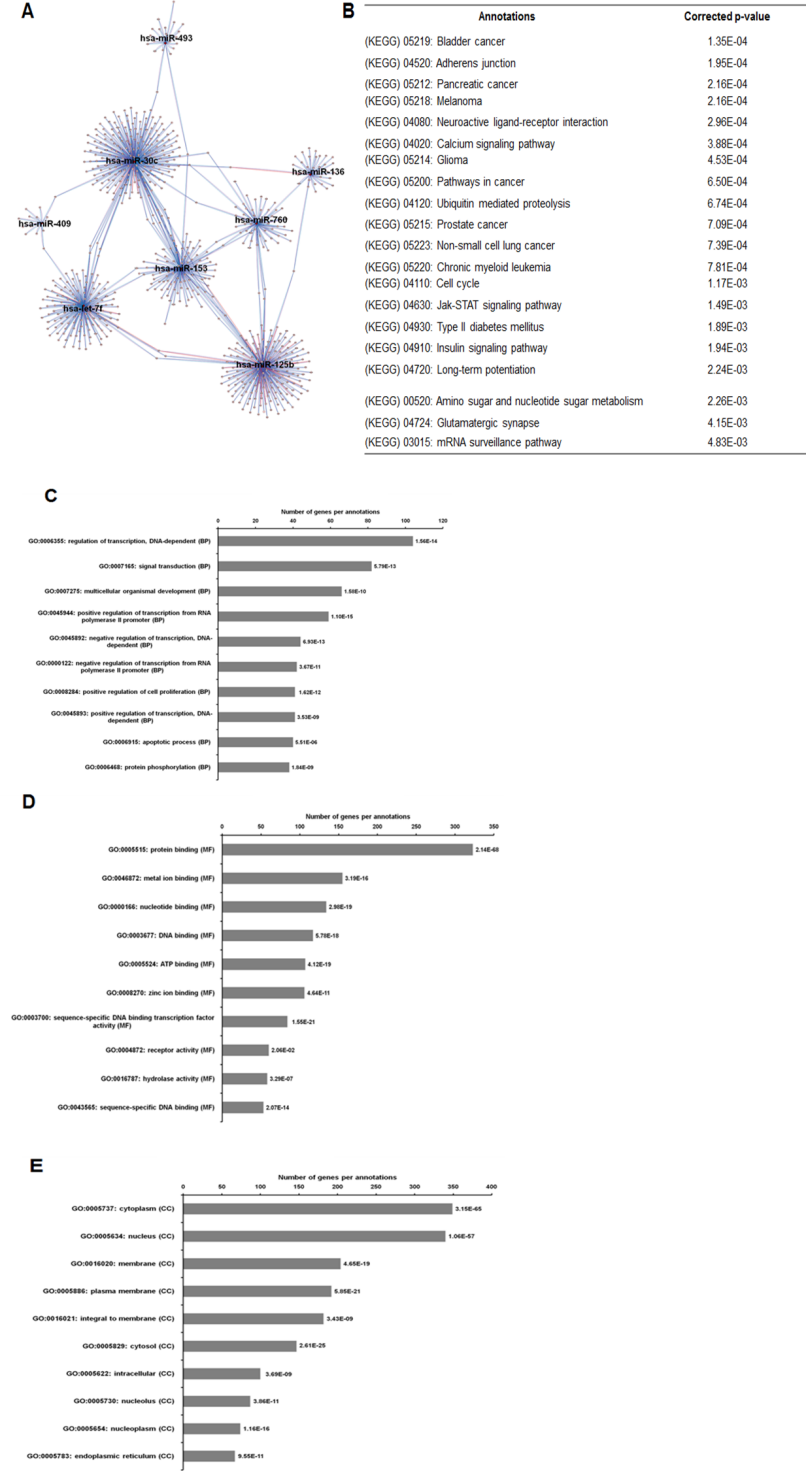
The network, pathway analysis and functional annotation by GO for predicted miRNAs targets exclusively expressed in MCF-7 spheroid-enriched CSCs cells. (A) Network of differentially expressed miRNAs-target genes. Each network includes two types of nodes, red circle represents individual miRNAs and pink circle represents target genes. (B) List of enriched KEGG pathways with their corresponding corrected *p*-values based on hypergeometric test with the most significant pathways (smallest *p*-value) listed from top down. Gene ontology analysis in the (C) biological process, (D) in molecular functions, and (E) in cellular components. The number of genes per annotations indicating enriched levels of genes with a modified Fisher exact *P*-value <0.05 are shown at the end of the nodes.

**Figure 7:**
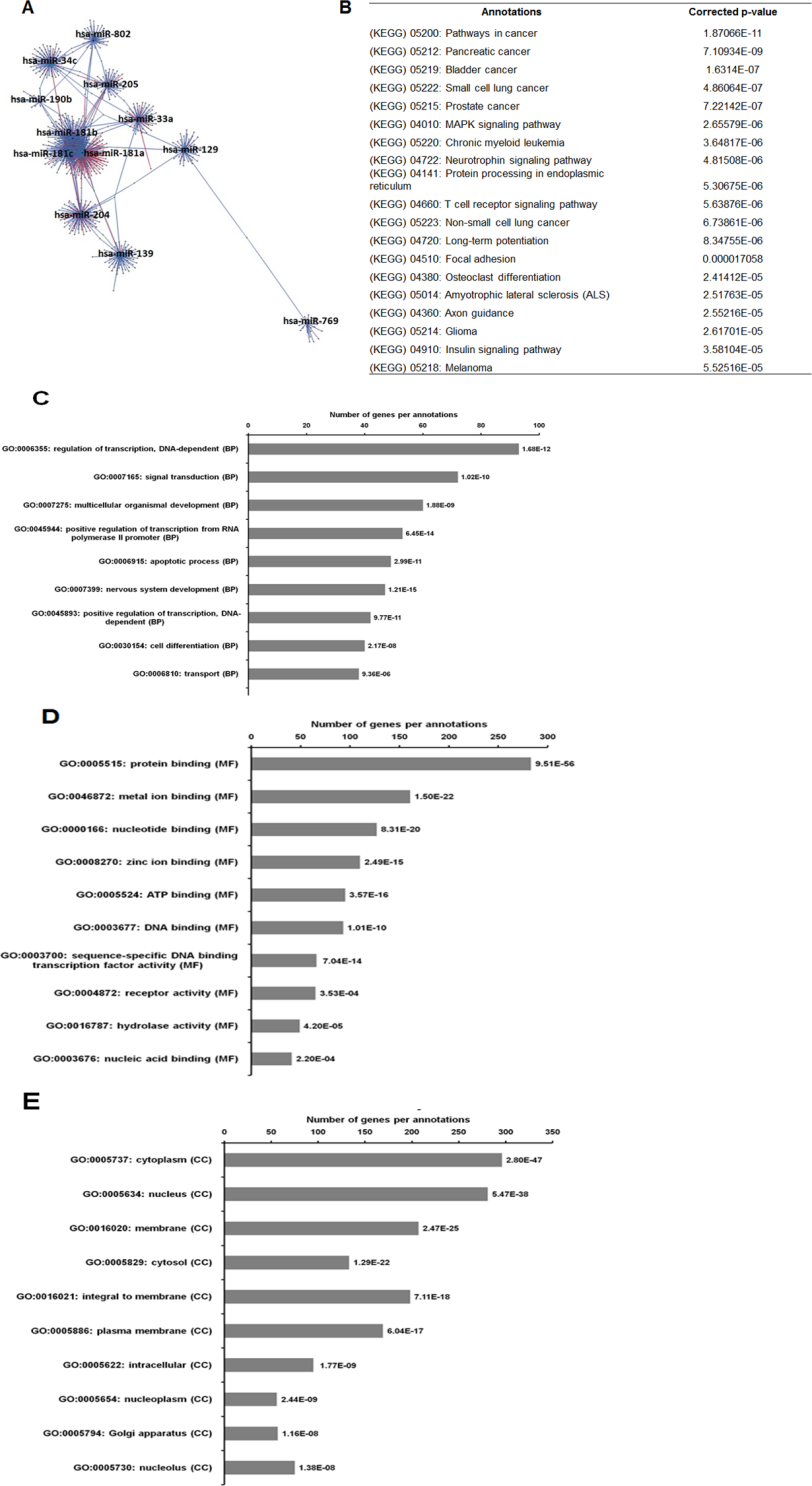
The network, pathway analysis and functional annotation by GO for predicted miRNAs targets exclusively expressed in MDA-MB-231 spheroid-enriched CSCs cells. (A) Network of differentially expressed miRNAs-target genes. Each network includes two types of nodes, red circle represents individual miRNAs and pink circle represents target genes. (B) List of enriched KEGG pathways with their corresponding corrected *p*-values based on hypergeometric test with the most significant pathways (smallest *p*-value) listed from top down. Gene ontology analysis in the (C) biological process, (D) in molecular functions, and (E) in cellular components. The number of genes per annotations indicating enriched levels of genes with a modified Fisher exact *P*-value <0.05 are shown at the end of the nodes.

As illustrated in Fig. 5, 102 deregulated miRNAs (15 up and 87 down) were found only in MCF-7 spheroid, while 46 deregulated miRNAs (20 up and 26 down) were exclusively found in MDA-MB-231 spheroid. Most of the deregulated miRNAs observed in both spheroid cells (87 miRNAs in MCF-7 spheroid while 26 in MDA-MB-231 spheroid) were all down-regulated, suggesting that most of the miRNAs in the spheroid-enriched breast cancer cells may be tumour suppressors. Besides, our results demonstrated that a complex network of the cluster of miRNAs and their predicted target genes were spheroid-breast cancer subtype specific. The miRNAs exclusively found in MCF-7 spheroid cells were evaluated for their target gene predictions. Gene annotations indicated that the target genes inferred from the group of deregulated miRNAs were associated with various biological characteristics like regulation of transcription factors, and signal transduction; molecular functions including protein binding and metal ion binding; and the gene products were primarily found in cell cytoplasm, membrane and nucleus (Fig. 6). The enriched pathways as revealed by Kyoto Encyclopaedia of Genes and Genomes (KEGG) analysis, includes JAK-STAT signalling pathway, calcium signalling and insulin signalling pathway. Meanwhile, the group of deregulated miRNAs found only in MDA-MB-231 spheroid targeted genes had the similar enriched Gene Ontology (GO) (Fig. 7). To our surprise, the enriched KEGG pathways were also found to be quite similar to those found in MCF-7 spheroid. This implied that although different cluster of miRNAs were expressed in MCF-7 and MDA-MB-231 exclusively in spheroid-enriched CSCs, the enriched gene sets and their associated pathways were similar. Nevertheless, miRNAs expressed exclusively in MDA-MB-231 spheroid that were associated with neurotrophin signalling pathway, p53 signalling pathway and ECM-receptor interaction were chosen for the validation purpose as these signalling pathways have shown potential for cancer targeted therapy (Hondermarck, 2012; Liu et al., 2015; Wang & Sun, 2009).

### Common pathways in MCF-7 and MDA-MB-231 spheroids linked to CSCs-related signalling pathways

**Figure 8:**
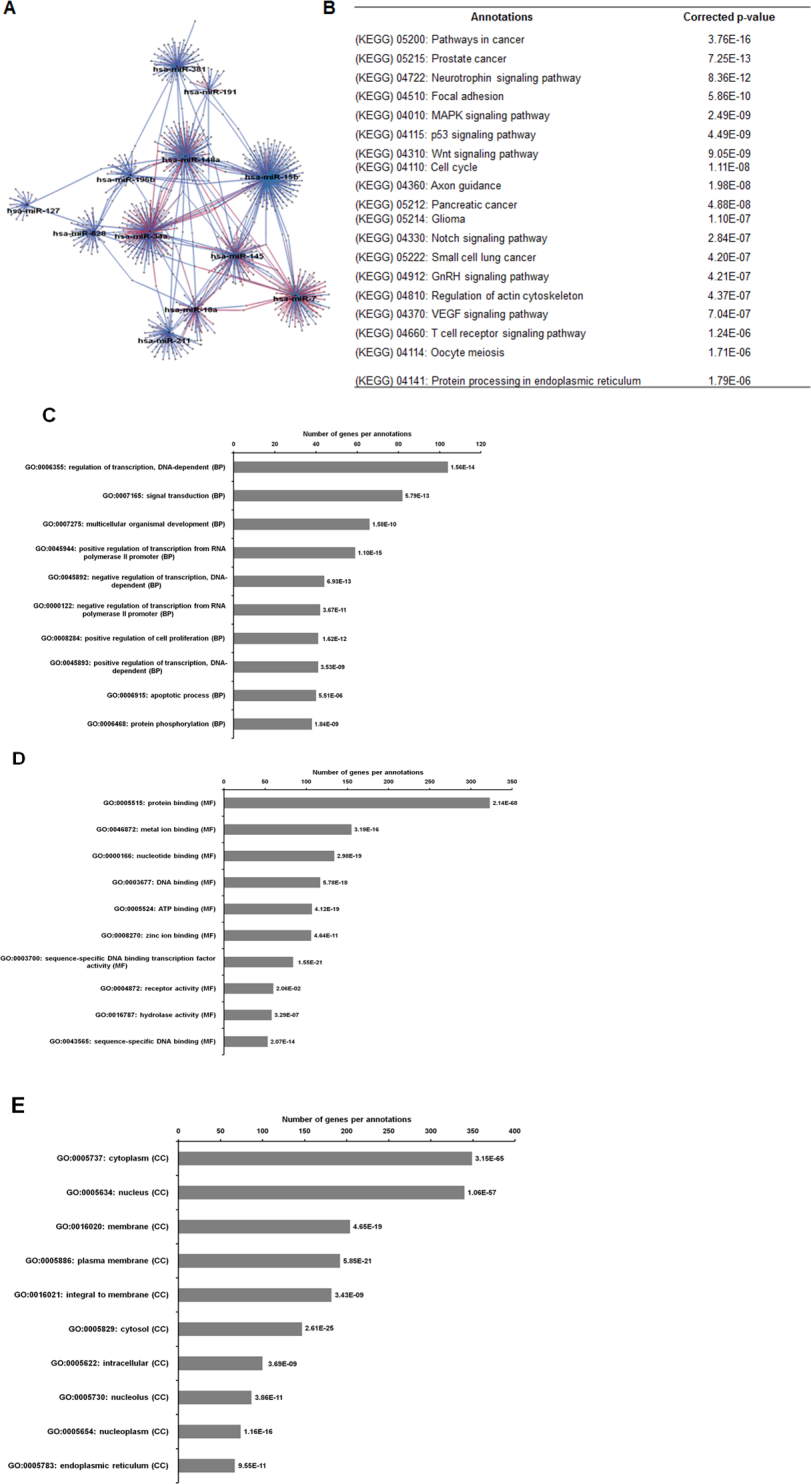
The network, pathway analysis and functional annotation by GO for predicted miRNAs targets commonly expressed in the two spheroid-enriched CSCs cell types. (A) Network of differentially expressed miRNAs-target genes. Each network includes two types of nodes, red circle represents individual miRNAs and pink circle represents target genes. (B) List of enriched KEGG pathways with their corresponding corrected *p*-values based on hypergeometric test with the most significant pathways (smallest *p*-value) listed from top down. Gene ontology analysis in the (C) biological process, (D) in molecular functions, and (E) in cellular components. The number of genes per annotations indicating enriched levels of genes with a modified Fisher exact *P*-value <0.05 are shown at the end of the nodes.

A total of 20 miRNAs were found to be commonly deregulated between these two breast cancer spheroid-enriched CSCs cell types, as illustrated in Fig. 5. We further explored the targets genes using Cytoscape which utilizes three target prediction algorithms in order to obtain more confident target genes for the cluster of miRNAs. Interestingly, when the target genes were inferred from the deregulated common group of miRNAs between the two spheroid models, they were found to be significantly positively regulated in processes such as transcription factors, cell proliferation, and molecular functions in nucleotide binding, DNA binding and protein kinase binding (Fig. 8). Among the commonly expressed validated miRNAs, miR-15b-5p, miR-34a-5p and miR-148a-5p were with the most target genes. The enriched genes were involved in pathways commonly found in cancer and stem cells primarily on Focal adhesion, MAPK, Wnt, Notch, Hedgehog, mTOR, and VEGF. Further validation was conducted on the five miRNAs representing the target genes associated with these pathways using qRT-PCR expressions analysis.

### Quantitative PCR validation

Twelve known miRNAs representing specific pathways found in MCF-7 and MDA-MB-231 spheroid-enriched CSCs were picked for qRT-PCR (Data S6). The miRNAs assessed were hsa-miR-15b, hsa-miR-34a-5p, hsa-miR-148a-3p, hsa-miR-628-5p, and hsa-miR-196b (commonly expressed in both spheroids), hsa-miR-125b-5p, hsa-miR-760, hsa-miR-30c-5p, and hsa-miR-136-5p (exclusively expressed in MCF-7 spheroid) and hsa-miR-204-5p, hsa-miR-181a-5p and hsa-miR-205-5p (exclusively expressed in MDA-MB-231 spheroid). Utilising qRT-PCR, the levels of the selected miRNAs expressions in spheroid cells in relation to the parental cells were found to have consistent expression levels, suggesting that the miRNAs profiling from Illumina sequencing run were reliable (Data S7).

### Reversal spheroid cells retained CSCs characteristics

When subjected to routine monolayer culture condition, cells were seen to have attached to the plate and migrated out from the spheroids which eventually grew into monolayer cells based on microscopy examination. The levels of the selected miRNAs expression in the reversal spheroid cells were shown to express similar patterns to that of the spheroid cells (Data S9).

## Discussion

A vast number of literatures including one of our recently published paper have demonstrated the successful application of serum-free enrichment technique to enrich for CSCs population (Boo et al., 2016; Fan et al., 2010). Using the similar methodology, we initiated spheroid cells from MDA-MB-231 breast cancer cell lines, which is one of the commonly used TNBC cell line (Chavez, Garimella & Lipkowitz, 2010). Recent advancement in NGS and bioinformatics analysis has provided the opportunity to explore the associated miRNA profiling in the spheroid cell models as a guide for future identification of potential miRNAs markers in breast CSCs studies (Shyr & Liu, 2013).

In our current study, MDA-MB-231 spheroids were found to demonstrate typical CSCs characteristics, namely, self-renewability, overexpressing certain stem cell-associated surface and intracellular markers, increased expression of CD44+ and ALDH+ and also increased resistance to chemotherapeutics drugs, which is in agreement with other studies (Lopez et al., 2012; Piero, Robert & Michael, 2007). When the biological properties of these two spheroid models were compared, both spheroids were capable of forming secondary spheroids under limiting dilution assay, but the morphological features between these cells were different. MDA-MB-231 secondary spheroid enlarged into approximately 380 µm while MCF-7 secondary spheroids estimated to be 303 µm in size after 14 days in culture. SFE analysis showed that dissociated single cells from MDA-MB-231 spheroids had a higher SFE rate as compared to MCF-7 spheroids. When the CD44+/CD24− and ALDH+ expression were compared between the two spheroids, MDA-MB-231 spheroids displayed a higher fraction of cells displaying those markers. This is expected as it has been demonstrated that basal-subtypes of breast cancer, for instance, MDA-MB-231 cells are predominantly CD44+ (Fillmore & Kuperwasser, 2008). Yet compared to the parental cells, MDA-MB-231 exhibited higher ADLH expression, about 2-fold more than that of MCF-7 spheroids. Also, expression of ALDH in breast tumour cells has been correlated with higher pool of CSCs population (Douville, Beaulieu & Balicki, 2008). Thus, the MDA-MB-231 spheroids were enriched with higher CSCs population relative to MCF-7 spheroids. MDA-MB-231 which is characterised by their basal/mesenchymal phenotype has been shown to mask differentiation as compared to the increased expression of differentiation-associated genes found in luminal cell type. Therefore, it is postulated that MDA-MB-231 harbours a higher quiescent pool of CSCs (Akrap et al., 2016). In view of their drug resistance properties, both spheroids had higher drug inhibitory concentration relative to the parental cells. Interestingly, doxorubicin, a common chemotherapeutic drug against breast cancer, was more resistant in MCF-7 spheroids (18-fold) than in MDA-MB-231 spheroids (8-fold). MDA-MB-231 which formed a loosely and less compact spheroid than MCF-7, could be the reason behind the higher doxorubicin drug penetration which resulted in a lower drug inhibitory concentration. A similar trend was also reported with the cytotoxicity study of a ginger compound 6-shogaol in MCF-7 spheroids (Ray, Vasudevan & Sengupta, 2015).

In the concept of CSCs, some pathways are responsible for the induction of Epithelial-to-Mesenchymal Transition (EMT), maintaining stemness of CSCs as well as the increased levels of cell proliferation, invasion and migration that distinguish stem cells from non-stem cells (Takebe et al., 2015). A few potential targeting pathways have been previously identified to eliminate CSCs such as the altering the self-renewal mechanisms (Wnt, Notch, Hedgehog) or the inhibition of the tumour progression and metastasis (JAK-STAT, TGF-β, PDFGR) (Pattabiraman & Weinberg, 2014). Apart from these common pathways, new therapies targeting the complex biology of CSCs were also being elucidated (Shibuya et al., 2015).

Here, we address a cluster of potential miRNAs associated with the CSCs-signalling pathways that could aid in the development of therapeutic strategies targeting miRNAs for future CSCs therapy. Our study demonstrated that there were 20 significantly deregulated miRNAs that overlapped between these two spheroids models, suggesting that there are only a small portion of miRNAs are being shared by the spheroid models (Fig. 5). These miRNAs were involved in some crucial cancer-associated and stem cell regulation of pathways. The targets of miR-15b-5p were LRP6 and WNT5B, was shown to be related to Wnt signalling pathway, while the targets of miR-34a-5p were NOTCH-associated genes involved in Notch signalling pathway. As for miR-148a-5p, the targets were GAS1 and LRP2, which was related to Hedgehog signalling pathway. Interestingly, among this cluster of commonly expressed miRNAs identified, miR-34a-5p was previously implicated in colon, pancreatic and prostate cancer modulating CSCs self-renewal or cell fate determination where it directly targets Notch receptors (Bu et al., 2013; Li, Ren & Tang, 2014). As such, these spheroid-enriched CSCs inducible miRNAs may have considerable clinical value in CSCs diagnosis (Morata-Tarifa et al., 2016). Besides being involved in self-renewal pathway, both miR-15b-5p and miR-34a-5p were associated with MAPK signalling pathway and miR-628-5p were predicted to be involved in mTOR signalling pathway (Hua et al., 2006; Matter et al., 2014; Wu, Yang & Li, 2009). However, the function of miR-196b-5p in breast cancer is still uncertain. Our study showed that miR-196b-5p was down-regulated in both spheroid subtypes while another study showed that miR-196b-5p was specifically up-regulated leading to reduction of apoptosis in colorectal cancer cells by mediating FAS gene expression (Mo et al., 2015). The conflicting results might be attributed by the genetics of the different cell lines used and also the targeted genes which are yet to be validated. Nevertheless, it is worth exploring the other afore-mentioned miRNAs that are involved in the other stem cell-related signalling pathways. Modulation of this cluster of miRNAs could be a promising approach to suppress those pathways, thus improving the effectiveness of breast cancer treatments. When subjected to reverse culture, the migrated spheroid cells reverted to their monolayer characteristics which could be due to the serum culture condition that facilitated the attachment, migration and propagation of the reversal cells. However, those cells still maintain the selected miRNA expression pattern as that of the spheroid cells. Nevertheless, more work must be carried out which include isolation of individual population of the cells to confirm the involvement of the differentially expressed miRNAs in the maintenance of the spheroid cells and their CSC-like properties.

In the current study, when we compared the miRNAs profiling of the MDA-MB-231 spheroid cells to the MCF-7 spheroid cell, we found a set of miRNAs exclusively expressed either in MDA-MB-231 or MCF-7 spheroid cells (Fig. 5). A considerable amount of the differentially expressed miRNAs that were exclusively found in MDA-MB-231 spheroid (46 miRNAs) and in MCF-7 spheroid (102 miRNAs) were attributed by the fact that they were two distinct breast cancer subtypes. For instance, miR-205-5p preferentially expressed in basal-like breast cancer cells is normally found in triple negative breast tumours, is a key player in cell cycle progression whereby it regulates cell proliferation, cell progression and enhance metastasis (Elgamal et al., 2013; Radojicic et al., 2011). Similarly, miRNA (miR-181a-5p) which was commonly found in TNBC tissues was also found to be upregulated in the MDA-MB-231 spheroids (Taylor et al., 2012) was reported to inhibit TGFβR3 protein translation. Subsequently, this inhibtion increases metastasis, invasion, migration, and reverting anoikis resistance in TNBC through their negative regulation in TGF-beta signalling pathway. miR-204-5p which has been reported as down-regulated in a few cancers (Yin et al., 2014), was also present at low levels in theMDA-MB-231 spheroid and their reduced expression of miR-204-5p is associated with poor clinical outcome in basal-like TNBC, suggesting their potential diagnostic use (Li et al., 2014). In contrast, ectopic expression of miR-204-5p has been demonstrated to restore anoikis sensitivity and reduced the invasiveness and metastatic behaviour in ovarian cancer cell line (Yan et al., 2015). Therefore, targeting miR-204-5p could possibly enhance anoikis sensitivity of basal-like breast cancer via neutrotrophin signalling pathway, thus making it a possible marker for metastasis breast cancer. Based on the findings from these results and on previous literature, the regulation of this cluster of miRNAs may play important roles in the metastatic and chemoresistance abilities of basal-like spheroid cancer cells.

On the other hand, we identified four miRNAs (miR-125b-5p, miR-760, miR-30c and miR-136-5p) exclusively expressed in MCF-7 spheroids based on their potential roles in luminal-like breast tumorigenesis via regulation of a number of predicted cancer-associated pathways including JAK-STAT, calcium, and insulin signalling pathways. Among the down-regulated miRNAs, miR-125b-5p was favoured to have tumour suppressor roles by targeting ENPEP gene, where enforced expression in MCF-7 cells reduced cell proliferation and anchorage-independent growth by positive regulation in breast tumorigenesis (Feliciano et al., 2013). Downregulated expression of miR-125b-5p was also found to increase HER2 protein expression, leading to worse prognostic outcome in luminalA breast cancer patients (Bailey, Westerling & Brown, 2015). Moreover, deregulated expression of miR-760 was previously reported as one of the miRNAs regulated by estrogen-responsive gene clusters, suggesting its potential use as biomarkers for the luminal-like breast cancer cells (Cicatiello et al., 2009). In another similar study, expression of miR-30c has been linked to chemotherapy resistance specifically in luminal A breast tumours by regulating TWF1 and IL-11 genes in the miR-30c-mediated pathway (Bockhorn et al., 2013) and deregulation of miR-30c has led to increase in cell proliferation, drug sensitivity and cancer progression. As for miR-136-5p, although it has not been to be associated with luminal-like spheroid breast cancer, it previously reported to be deregulated in lung cancer (Shen et al., 2013) and ovarian cancer (Zhao et al., 2015), suggesting the potential use as biomarkers in this subtype of breast cancer.

Overall, the unique cluster of miRNAs found in each cell type are associated with their chemoresistance properties and cancer progression, and most likely influencing the maintenance of the spheroid-enriched CSCs properties. Moreover, bioinformatics analysis revealed that the predicted gene annotation and pathways inferred from the uniquely expressed set of miRNAs found in the two breast spheroids models were relatively similar (Figs. 6B and 7B). This indicates the highly complex interactions of miRNAs in the regulation of gene expressions in which MCF-7 and MDA-MB-231 spheroid cells may use different miRNAs to regulate their biological properties, but those miRNAs converged to the similar pathways and gene products. Nonetheless, further validation of the miRNAs, their target genes and the relevant pathways can help researchers in designing and developing strategies of novel therapeutic in battling breast cancer.

## Conclusions

Here we have determined the CSCs characteristic and profiling of the MDA-MB-231 spheroid model. Compared to the previously reported miRNA profiling of the MCF-7 spheroid, we further demonstrated that the spheroid culturing method can be used to enrich for CSCs-like subpopulations in both breast cancer cell lines. The results confirmed that both spheroids expressed certain miRNAs unique to their cancer subtypes and also a cluster of miRNAs that were commonly expressed in both spheroids. This study enhances our understanding not only on the roles of miRNAs in spheroid-enriched CSCs models, but more importantly highlights the 20 commonly deregulated miRNAs found in both spheroids cells. To the best of our knowledge, no study has shown a comparison of the differentially expressed miRNAs from these two CSCs enriched spheroid models. As such, these miRNAs profiling datasets could be used as reference for future investigations on their role in spheroid CSCs enriched breast cancer models, leading to potentially more effective targeted breast cancer therapies which may provide insights of the miRNAs mechanism and relationship in the spheroid models.

##  Supplemental Information

10.7717/peerj.3551/supp-1 Data S1 Fluorescence images of spheroids and monolayer cells Immunofluorescent staining of CSCs-related surface and internal markers on spheroids and the monolayer cells (controls). DAPI was used for nuclear counterstain. Magnifications were at 4× and 10×.

10.7717/peerj.3551/supp-2 Data S2 Length distribution of miRNAs reads Arrows indicate the average size of the reads which are 22 nucleotides in size for the both parental (A) and spheroid MDA-MB-231 (B) cells.

10.7717/peerj.3551/supp-3 Data S3 Small-RNA library sequencing summary

10.7717/peerj.3551/supp-4 Data 4 Quality control of the miRNA-NGS data The differentially expressed miRNAs data was analysed using hierarchical clustering (A), principal component analysis (PCA) (B), and volcano plot (C) to interpret the differential expression pattern between groups. (A) Heat map shows the results of two-way hierarchical clustering of miRNAs and samples. (B) PCA plot on miRNA expression data from MDA-MB-231 spheroid and parental cells indicate the relative differential expression between groups. (C) Volcano plot showing significantly (Fold change >2.0 and *P*-values <0.05) differentially expressed miRNAs in MDA-MB-231 spheroid relative to parental cells. Dots in blue represent miRNAs that do not have significant changes in expression, while dots in red on the left indicate miRNAs with significantly down-regulated expression, and on the right indicates the miRNAs with significantly up-regulated expression.

10.7717/peerj.3551/supp-5 Data S5 Differentially expressed miRNAs List of differentially expressed miRNAs with a two or greater fold change in spheroids MDA-MB-231 relative to parental culture (FC >2, P <0.05).

10.7717/peerj.3551/supp-6 Data S6 Real-time PCR validation The list of miRNAs used for the qRT-PCR validation.The names of the miRNAs primers, sequence accession number and target sequences are shown.

10.7717/peerj.3551/supp-7 Data S7 Validation of miRNA-NGS results using qRT-PCR Comparison of the qRT-PCR and miRNA sequencing log2 fold change for 12 known miRNAs between parental and spheroid cells. (A) qRT-PCR analysis in MCF-7 spheroid relative to its parental cells (B) 3 qRT-PCR analysis in MDA-MB-231 spheroid relative to its parental cells. A similar expression was observed between qRT-PCR and NGS analysis.

10.7717/peerj.3551/supp-8 Data S8 Original figures of flow cytometric dot plots extracted from FACS Calibur

10.7717/peerj.3551/supp-9 Data S9 Characteristics of the reversal spheroid cells (A) Morphology of parental cells and induction of spheroid cells into monolayer culture condition for 5 days (magnification: 10×, scale bar; 100 µm). (B) qRT-PCR analysis in spheroid and reversal spheroid cells relative to the parental cells. A similar expression trend was observed between the both types of cells.

10.7717/peerj.3551/supp-10 Supplemental Information 1 Repository metadata GSE75396
METADATA spreadsheet submitted to GEO accession number GSE75396. All raw data can be found under the accession number.

10.7717/peerj.3551/supp-11 Data S10 Raw data

## Additional Information and Declarations

### Data Availability

The following information was supplied regarding data availability: The raw data has been provided as Supplementary File. NCBI: GSE75396.
