# Supplementary figures and images for: Phenotypic and microRNA transcriptomic profiling of the MDA-MB-231 spheroid-enriched CSCs with comparison of MCF-7 microRNA profiling dataset
Source: PeerJ. 2017 Jul 13;5:e3551. doi: 10.7717/peerj.3551 (PMC5511503; doi:10.7717/peerj.3551)

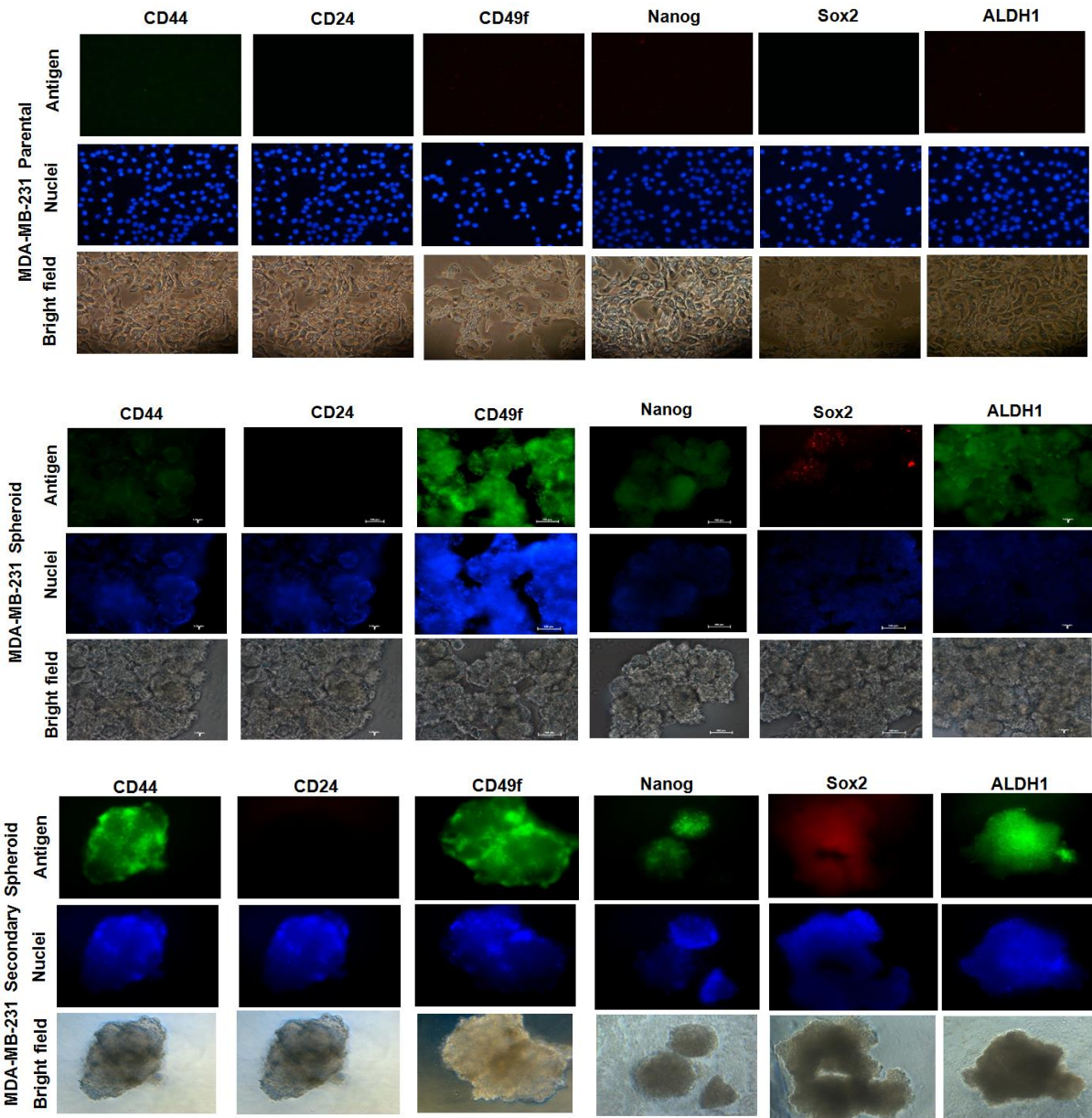

Supplement: Data S1 — Immunofluorescent staining of CSCs-related surface and internal markers on spheroids and the monolayer cells (controls). DAPI was used for nuclear counterstain. Magnifications were at 4× and 10×. [file peerj-05-3551-s001.pdf]

**A**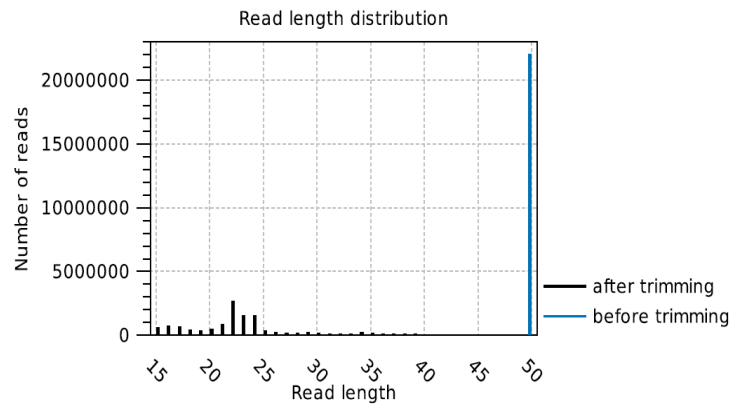**B**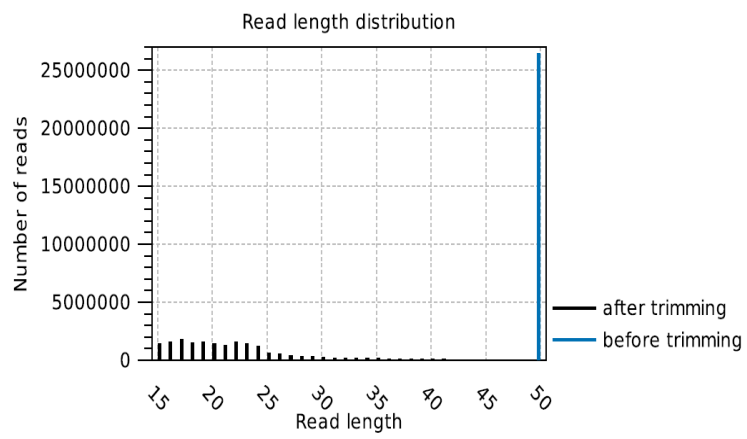

Supplement: Data S2 — Arrows indicate the average size of the reads which are 22 nucleotides in size for the both parental (A) and spheroid MDA-MB-231 (B) cells. [file peerj-05-3551-s002.pdf]

A

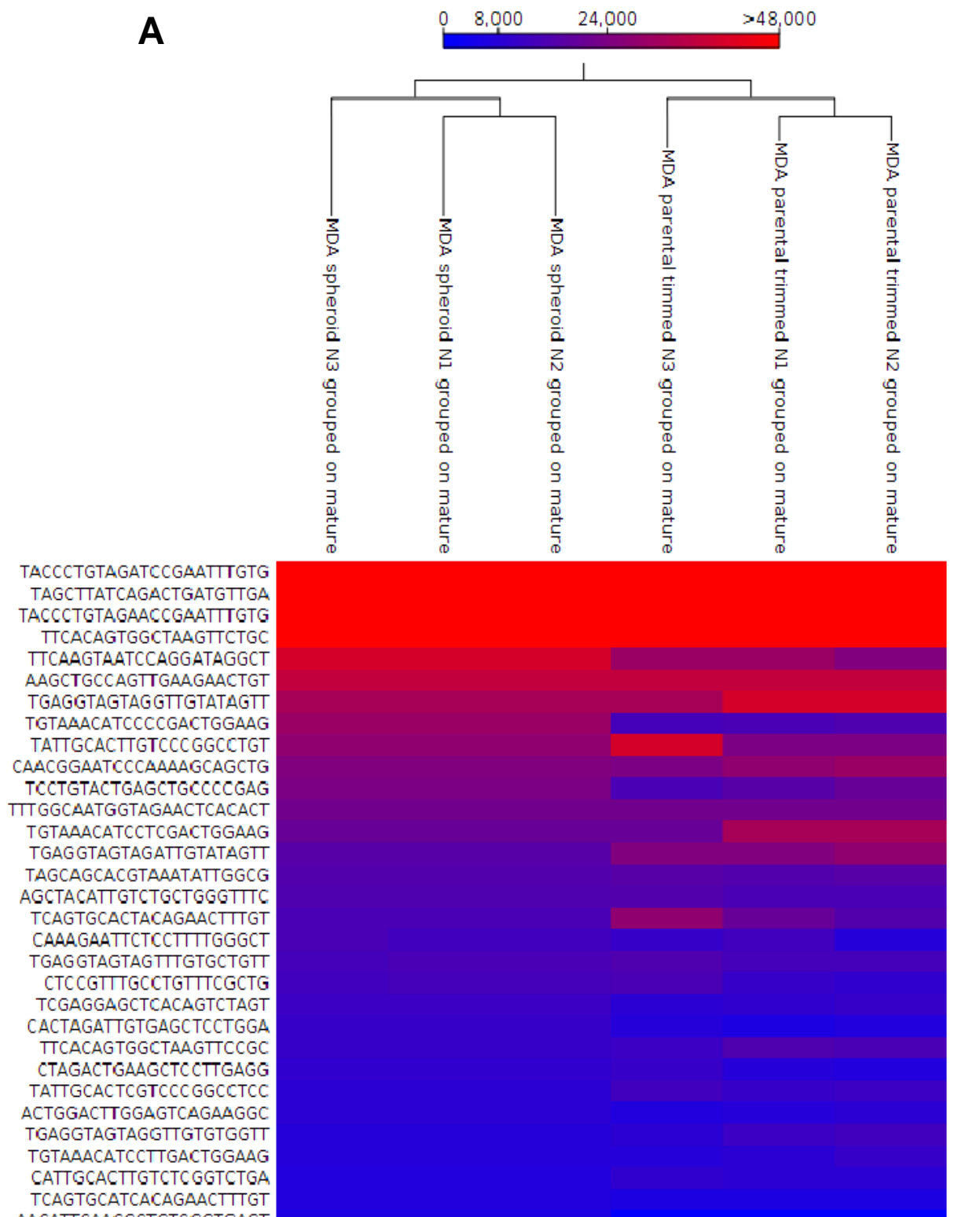

**B**

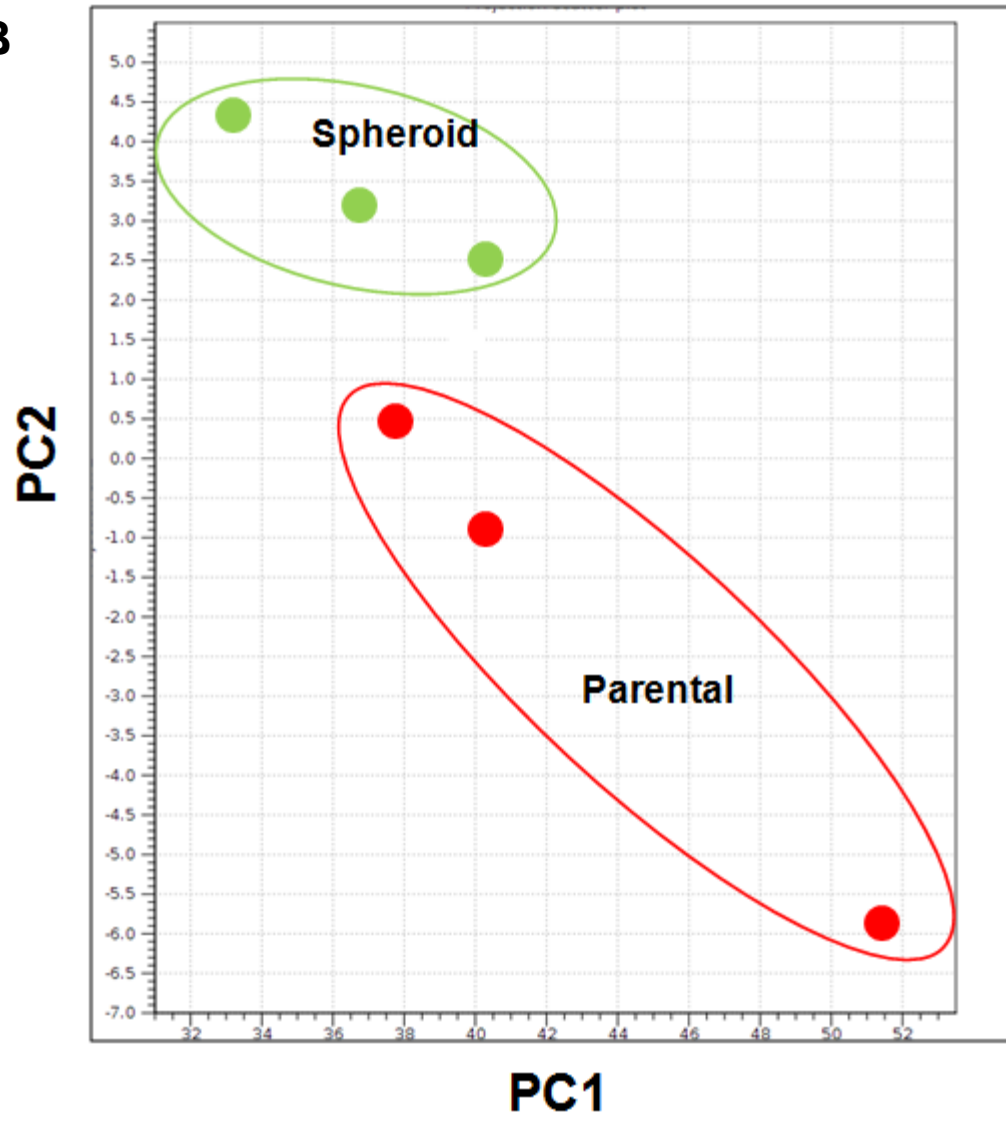

C

## Volcano Plot (Kal's test)

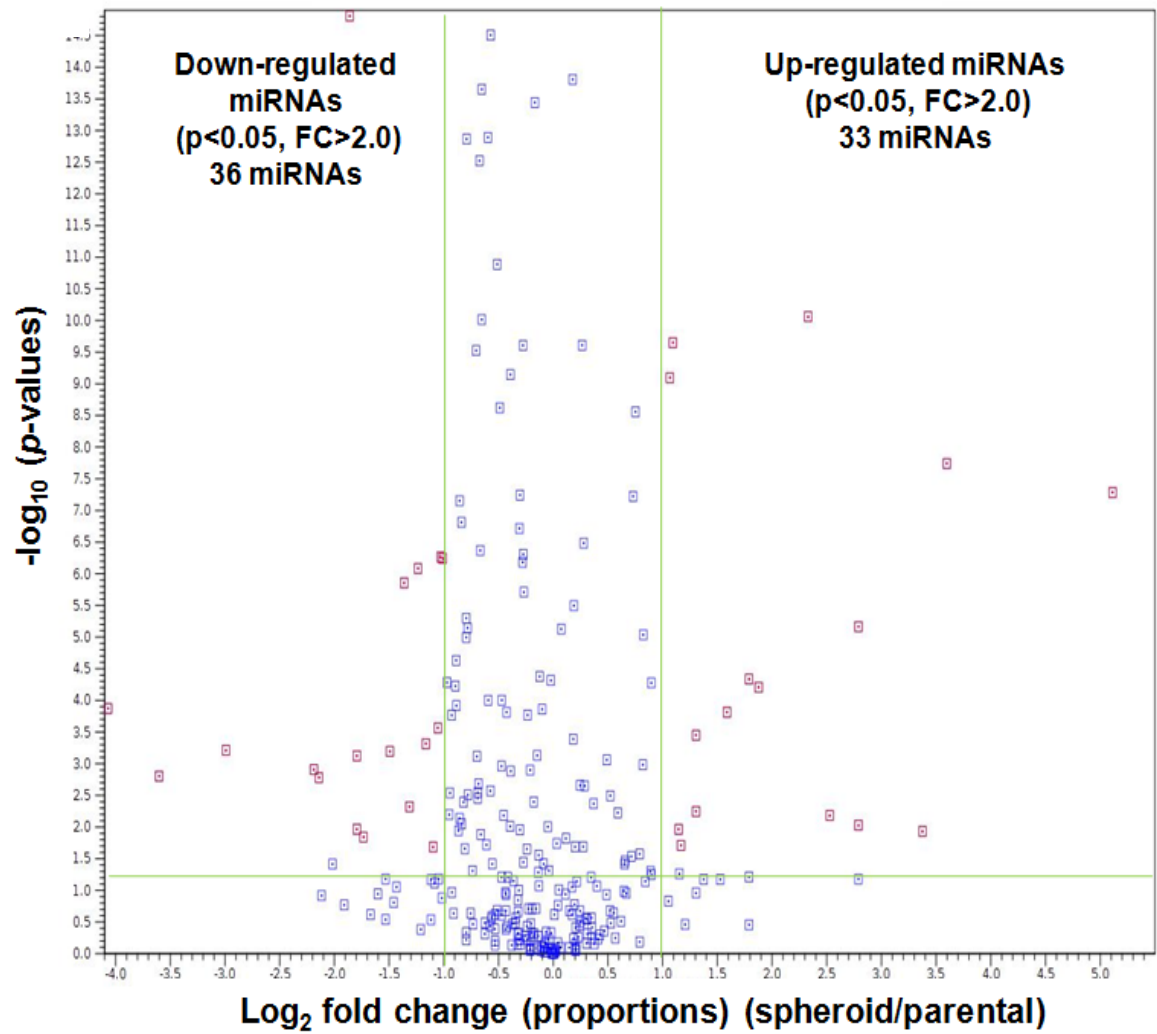

Supplement: Data 4 — The differentially expressed miRNAs data was analysed using hierarchical clustering (A), principal component analysis (PCA) (B), and volcano plot (C) to interpret the differential expression pattern between groups. (A) Heat map shows the results of two-way hierarchical clustering of miRNAs and samples. (B) PCA plot on miRNA expression data from MDA-MB-231 spheroid and parental cells indicate the relative differential expression between groups. (C) Volcano plot showing significantly (Fold change >2.0 and P-values <0.05) differentially expressed miRNAs in MDA-MB-231 spheroid relative to parental cells. Dots in blue represent miRNAs that do not have significant changes in expression, while dots in red on the left indicate miRNAs with significantly down-regulated expression, and on the right indicates the miRNAs with significantly up-regulated expression. [file peerj-05-3551-s004.pdf]

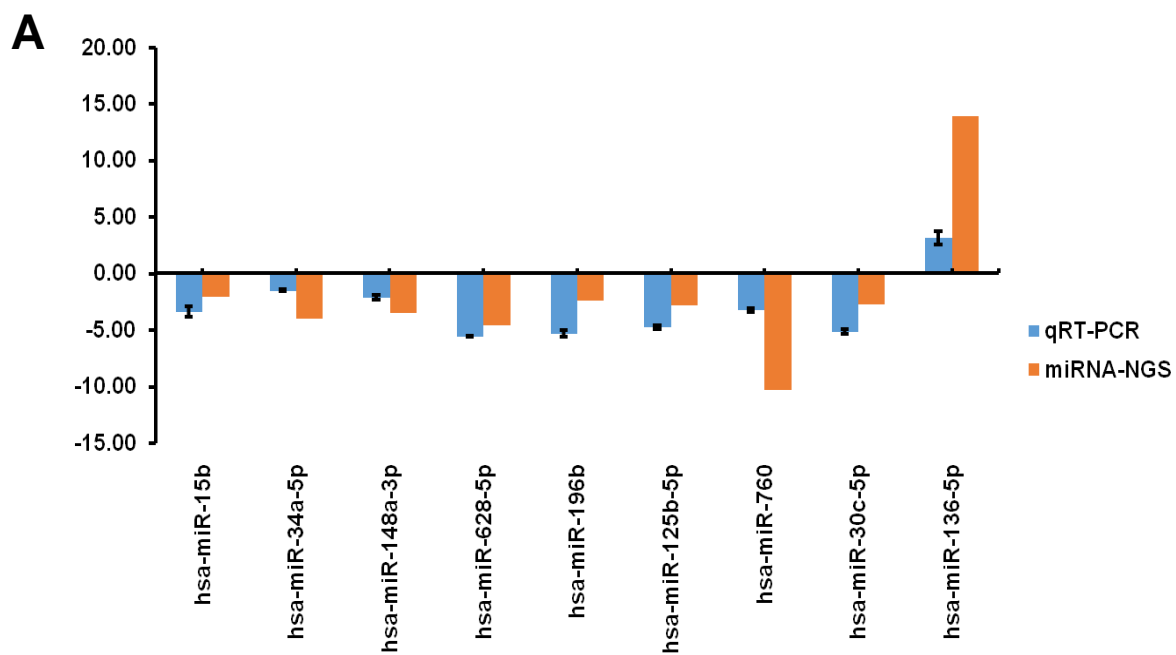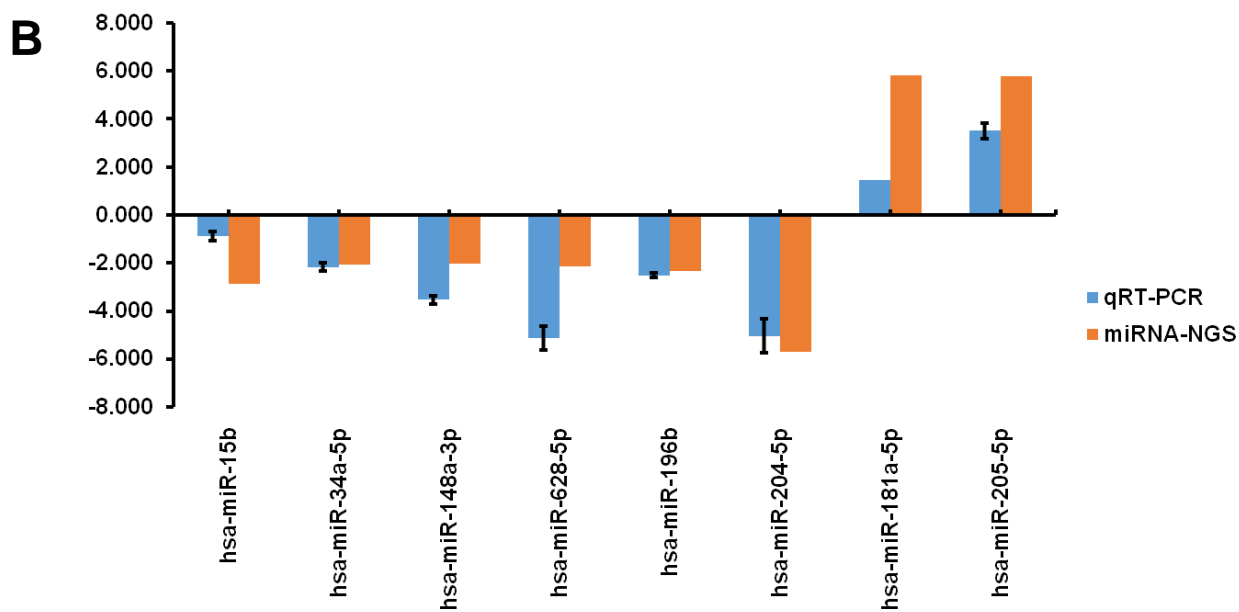

Supplement: Data S7 — Comparison of the qRT-PCR and miRNA sequencing log2 fold change for 12 known miRNAs between parental and spheroid cells. (A) qRT-PCR analysis in MCF-7 spheroid relative to its parental cells (B) 3 qRT-PCR analysis in MDA-MB-231 spheroid relative to its parental cells. A similar expression was observed between qRT-PCR and NGS analysis. [file peerj-05-3551-s007.pdf]

**A**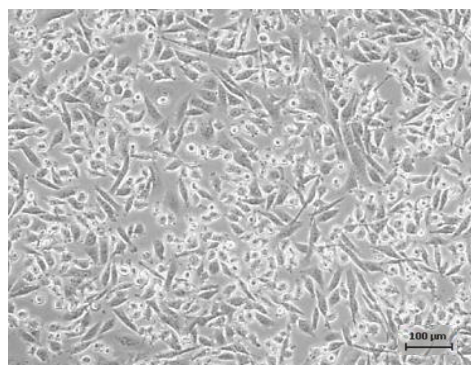**Parental**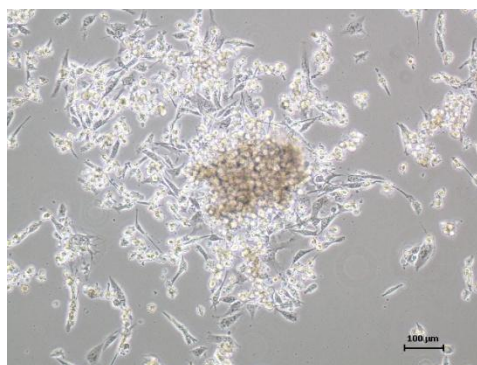**Reversal spheroid  
Day 3**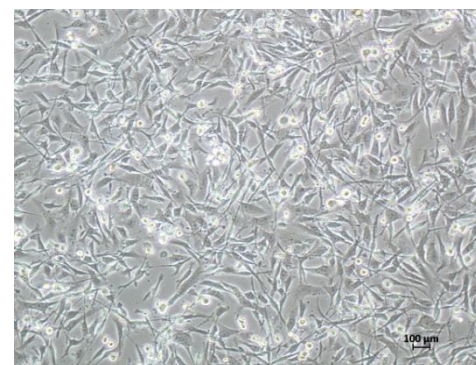**Reversal spheroid  
Day 5****B**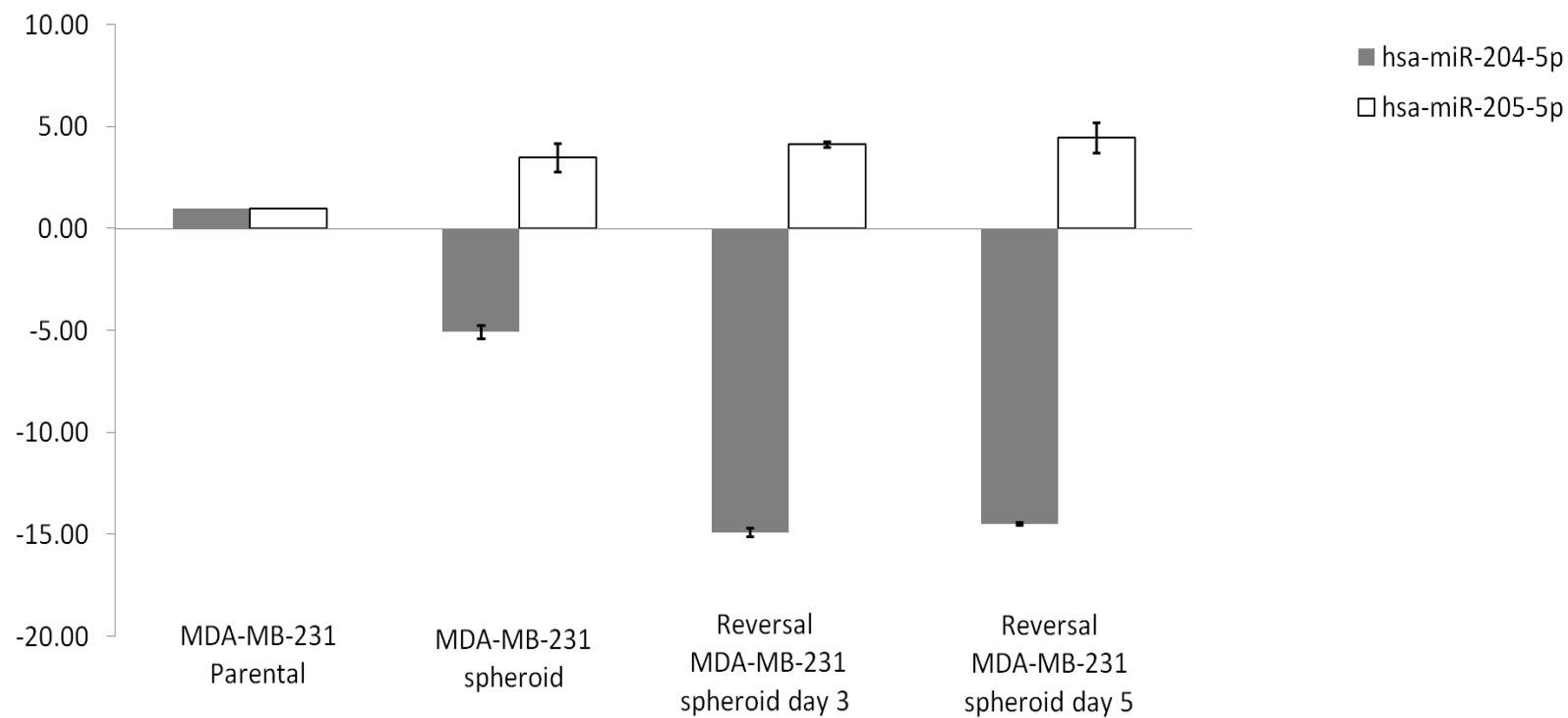

Supplement: Data S9 — (A) Morphology of parental cells and induction of spheroid cells into monolayer culture condition for 5 days (magnification: 10×, scale bar; 100 µm). (B) qRT-PCR analysis in spheroid and reversal spheroid cells relative to the parental cells. A similar expression trend was observed between the both types of cells. [file peerj-05-3551-s009.pdf]
