# Supplementary material for: Phenotypic and microRNA transcriptomic profiling of the MDA-MB-231 spheroid-enriched CSCs with comparison of MCF-7 microRNA profiling dataset
Source: PeerJ. 2017 Jul 13;5:e3551. doi: 10.7717/peerj.3551 (PMC5511503; doi:10.7717/peerj.3551)
Supplement: Data S3 [file peerj-05-3551-s003.pdf]

Cell types	MDA-MB-231 Parental	MDA-MB-231 Spheroid
**Index tag sequence**	GTGAAA	GTGGCC
**Total reads**	8,435,715	10,979,357
**Reads mapped to miRBase stem-loop sequences**	5,523,369	1,592,753
**Reads mapped to GRch37.57.ncrna**	1,132,822	171,524
**Unique sequences (unannotated)**	1,779,524	9,215,080
**Most abundant miRNA**	miR-21(108885)	miR-10a (277306)
**Total known miRNA**	54,042	29,260
