# Supplementary material for: Phenotypic and microRNA transcriptomic profiling of the MDA-MB-231 spheroid-enriched CSCs with comparison of MCF-7 microRNA profiling dataset
Source: PeerJ. 2017 Jul 13;5:e3551. doi: 10.7717/peerj.3551 (PMC5511503; doi:10.7717/peerj.3551)
Supplement: Data S5 — List of differentially expressed miRNAs with a two or greater fold change in spheroids MDA-MB-231 relative to parental culture (FC >2, P <0.05). [file peerj-05-3551-s005.pdf]

**miRNAs**	**Kal's Z-test p-value**	**Fold change>2 (Spheroid/Parental)**
mir-411	0.0000	41.495
mir-127	0.0000	33.838
mir-143	0.0000	17.732
mir-133a-1//mir-133a-2	0.0000	12.103
mir-410	0.0117	10.374
mir-145	0.0117	10.374
mir-126	0.0000	7.060
mir-1246	0.0000	6.916
mir-342	0.0094	6.916
mir-181a-1	0.0000	5.810
mir-205	0.0066	5.763
mir-210	0.0000	5.676
mir-139	0.0000	5.030
mir-181a-2//mir-181a-1	0.0000	5.019
mir-210	0.0000	3.716
mir-211	0.0001	3.674
mir-181b-1//mir-181b-2	0.0000	3.582
mir-181c	0.0000	3.458
mir-126	0.0000	3.429
mir-199a-1//mir-199a-2//mir-199b	0.0000	3.312
mir-1260a	0.0000	3.153
mir-1260b	0.0000	2.531
mir-365b	0.0004	2.470
mir-1291	0.0057	2.470
mir-885	0.0000	2.256
mir-885	0.0195	2.248
mir-664b	0.0109	2.213
mir-129-2	0.0000	2.134
mir-328	0.0000	2.094
mir-30d	0.0000	2.059
mir-423	0.0000	2.058
mir-191	0.0000	-2.014
mir-148a	0.0000	-2.034
mir-147b	0.0000	-2.040
mir-374b	0.0003	-2.078
mir-34a	0.0000	-2.079
mir-24-1//mir-24-2	0.0000	-2.109
mir-628	0.0207	-2.140
mir-10b	0.0005	-2.241
mir-196b	0.0000	-2.321

mir-449a	0.0000	-2.324
mir-345	0.0000	-2.355
mir-18a	0.0000	-2.504
mir-6087	0.0000	-2.571
mir-33b	0.0000	-2.582
mir-190a	0.0000	-2.593
let-7a-1//let-7a-3	0.0000	-2.612
mir-93	0.0006	-2.813
mir-15b	0.0000	-2.874
mir-184	0.0000	-2.892
mir-138-2//mir-138-1	0.0000	-2.958
mir-574	0.0000	-3.218
mir-802	0.0144	-3.326
mir-222	0.0005	-3.404
mir-1271	0.0000	-3.447
mir-200a	0.0108	-3.470
mir-24-1	0.0007	-3.470
mir-33a	0.0000	-3.631
mir-503	0.0017	-4.410
mir-34c	0.0012	-4.555
mir-204	0.0000	-5.692
mir-3613	0.0000	-6.423
mir-7-1//mir-7-2//mir-7-3	0.0000	-6.941
mir-769	0.0006	-7.953
mir-190b	0.0016	-12.146
mir-3074	0.0001	-16.773
