# Supplementary material for: Phenotypic and microRNA transcriptomic profiling of the MDA-MB-231 spheroid-enriched CSCs with comparison of MCF-7 microRNA profiling dataset
Source: PeerJ. 2017 Jul 13;5:e3551. doi: 10.7717/peerj.3551 (PMC5511503; doi:10.7717/peerj.3551)
Supplement: Data S6 — The list of miRNAs used for the qRT-PCR validation.The names of the miRNAs primers, sequence accession number and target sequences are shown. [file peerj-05-3551-s006.pdf]

Name	Sequence accession number	Target Sequence
hsa-miR-19a-3p (Reference)	MIMAT0000073	UGUGCAAAUCUAUGCAAAACUGA
hsa-miR-200a-3p (Reference)	MIMAT0000682	UAACACUGUCUGGUAACGAUGU
hsa-miR-15b-5p	MIMAT0000417	UAGCAGCACAUCAUGGUUUACA
hsa-miR-34a-5p	MIMAT0000255	UGGCAGUGUCUUAGCUGGUUGU
hsa-miR-148a-5p	MIMAT0004549	AAAGUUCUGAGACACUCCGACU
hsa-miR-628-5p	MIMAT0004809	AUGCUGACAUAUUUACUAGAGG
hsa-miR-196b-5p	MIMAT0001080	UAGGUAGUUUCCUGUUGUUGGG
hsa-miR-126-5p	MIMAT0000444	CAUUAUUACUUUUGGUACGCG
hsa-miR-760	MIMAT0004957	CGGCUCUGGGUCUGUGGGGA
hsa-miR-30c-5p	MIMAT0000244	UGUAAACAUCCUACACUCUCAGC
hsa-miR-136-5p	MIMAT0000448	ACUCCAUUUGUUUUGAUGAUGGA
hsa-miR-204-5p	MIMAT0000265	UUCCCUUUGUCAUCCUAUGCCU
hsa-miR-181a-5p	MIMAT0000256	AACAUUCAACGCUGUCGGUGAGU
hsa-miR-205-5p	MIMAT0000266	UCCUUCAUUCCACCGGAGUCUG
