# Supplementary material for: Phenotypic and microRNA transcriptomic profiling of the MDA-MB-231 spheroid-enriched CSCs with comparison of MCF-7 microRNA profiling dataset
Source: PeerJ. 2017 Jul 13;5:e3551. doi: 10.7717/peerj.3551 (PMC5511503; doi:10.7717/peerj.3551)
Supplement: Data S8 [file peerj-05-3551-s008.pdf]

MDA-MB-231 Parental

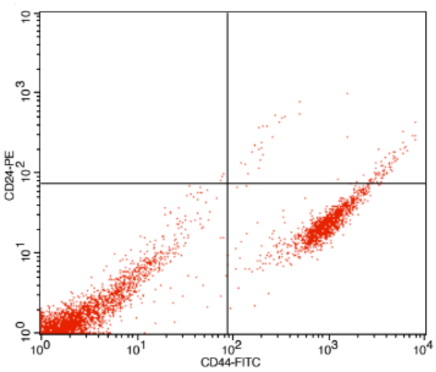

%Gated cell population
CD44+/CD24- = 27.21%

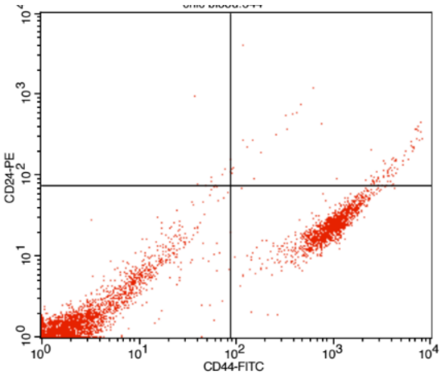

%Gated cell population
CD44+/CD24- = 28.96%

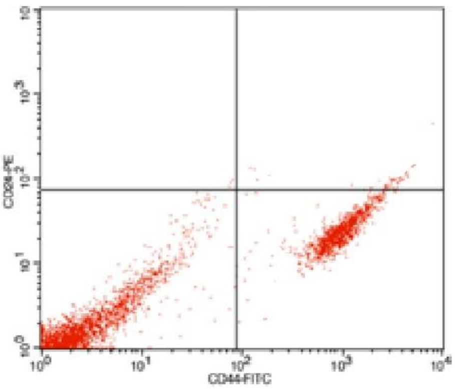

%Gated cell population
CD44+/CD24- = 25.66%

MDA-MB-231 Spheroid

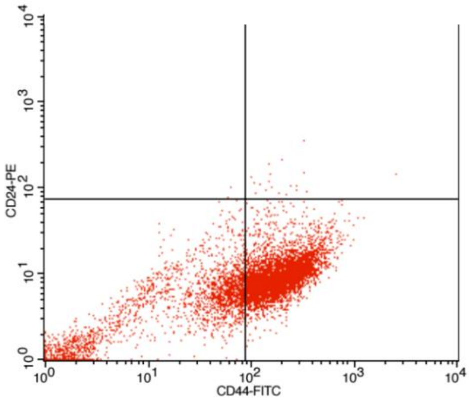

%Gated cell population
CD44+/CD24- = 68.63%

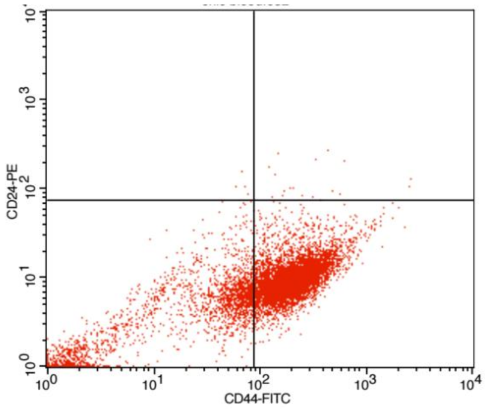

%Gated cell population
CD44+/CD24- = 72.90%

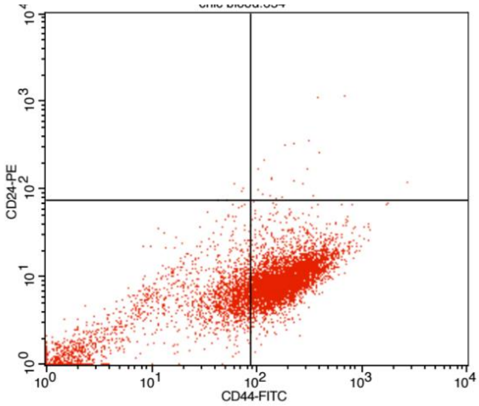

%Gated cell population
CD44+/CD24- = 69.72%
